# The Role of General Acid Catalysis in the Mechanism of an Alkyl Transferase Ribozyme

**DOI:** 10.1021/acscatal.4c04571

**Published:** 2024-10-02

**Authors:** Timothy J. Wilson, Erika McCarthy, Şölen Ekesan, Timothy J. Giese, Nan-Sheng Li, Lin Huang, Joseph A. Piccirilli, Darrin M. York, David M. J. Lilley

**Affiliations:** † Nucleic Acid Structure Research Group, Division of Molecular, Cellular and Developmental Biology, MSI/WTB Complex, The University of Dundee, Dow Street, Dundee DD1 5EH, U.K.; ‡ Laboratory for Biomolecular Simulation Research, Institute for Quantitative Biomedicine and Department of Chemistry and Chemical Biology, Rutgers University, Piscataway, New Jersey 08854, United States; § Department of Chemistry, The University of Chicago, Chicago, Illinois 60637, United States; ∥ Guangdong Provincial Key Laboratory of Malignant Tumor Epigenetics and Gene Regulation, Guangdong-Hong Kong Joint Laboratory for RNA Medicine, Sun Yat-sen Memorial Hospital, Sun Yat-sen University, Guangzhou 510120, P.R. China; ⊥ Medical Research Center, Sun Yat-sen Memorial Hospital, Sun Yat-sen University, Guangzhou 510120, P.R. China; # Department of Biochemistry and Molecular Biology, The University of Chicago, Chicago, Illinois 60637, United States

**Keywords:** RNA catalysis, catalytic mechanism, atomic mutagenesis, quantum mechanical calculation, general acid catalysis

## Abstract

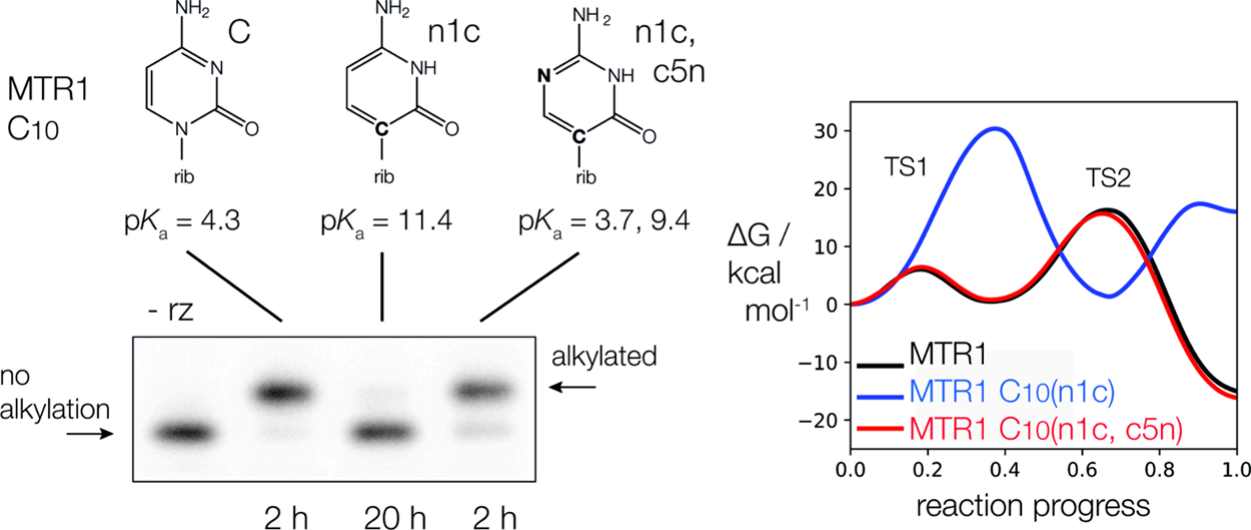

MTR1 is an in vitro-selected alkyl transferase ribozyme that transfers an alkyl group from *O*^6^-alkylguanine to N1 of the target adenine in the RNA substrate (A63). The structure of the ribozyme suggested a mechanism in which a cytosine (C10) acts as a general acid to protonate *O*^6^-alkylguanine N1. Here, we have analyzed the role of the C10 general acid and the A63 nucleophile by atomic mutagenesis and computation. C10 was substituted by n1c and n1c, c5n variants. The n1c variant has an elevated p*K*_a_ (11.4 as the free nucleotide) and leads to a 10^4^-fold lower activity that is pH-independent. Addition of the second c5n substitution with a lower p*K*_a_ restored both the rate and pH dependence of alkyl transfer. Quantum mechanical calculations indicate that protonation of *O*^6^-alkylguanine lowers the barrier to alkyl transfer and that there is a significantly elevated barrier to proton transfer for the n1c single substitution. The calculated p*K*_a_ values are in good agreement with the apparent values from measured rates. Increasing the p*K*_a_ of the nucleophile by A63 n7c substitution led to a 6-fold higher rate. The increased reactivity of the nucleophile corresponds to a β_nuc_ of ∼0.5, indicating significant C–N bond formation in the transition state. Taken together, these results are consistent with a two-step mechanism comprising protonation of the *O*^6^-alkylguanine followed by alkyl transfer.

## Introduction

The majority of contemporary ribozymes catalyze phosphoryl transfer reactions. However, the RNA world hypothesis requires that RNA could catalyze a wide variety of metabolic interconversion, including relatively “difficult” reactions including the formation of carbon–carbon and carbon–nitrogen bonds. The feasibility of RNA-catalyzed C–N bond formation has recently been demonstrated by the laboratory selection of a number of ribozymes that carry out methyl or alkyl transfer reactions, using a number of different alkyl donors.^1−4^ An association between methyl transfer and the RNA world is also suggested by the number of riboswitches in modern cells that bind methyl donors,^5−9^ and Flemmich et al.^10^ have shown that the prequeuosine1 (preQ1) riboswitch can exhibit a low rate of methyl transfer from *O*^6^-methyl preQ1 to N3 of cytosine in the RNA. A possible evolutionary relationship between riboswitches and RNA world ribozymes has been suggested previously.^11,12^

**Figure 1:**
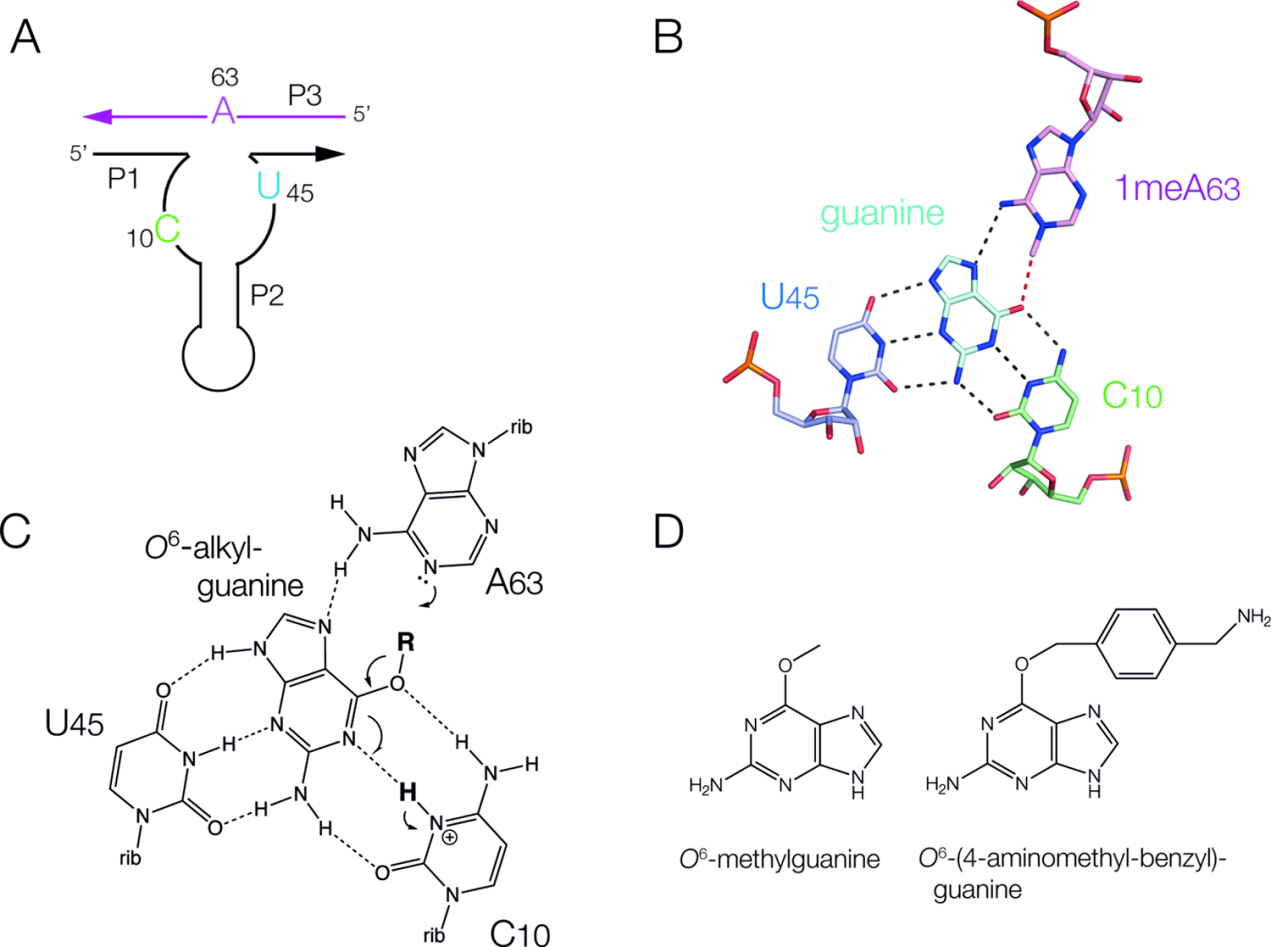
MTR1 alkyl transferase ribozyme. (A) The secondary structure of the MTR1 ribozyme, showing the key nucleotides. (B) The structure of the active center of the ribozyme from the crystal structure of the product complex.^13^ (C) A concerted mechanism for the alkyl transfer reaction as first proposed on the basis of the structure as observed in the crystal. The reaction involves transfer of a proton from C10 N3 to the N1 of the exogenous *O*^6^-alkylguanine, and nucleophilic attack by A63 N1 on the alkyl group. (D) Examples of substrates for alkyl transfer; *O*^6^-methyl guanine and *O*^6^-(4-aminomethyl-benzyl) guanine.

The MTR1 ribozyme was selected by Höbartner and co-workers^2^ to catalyze the transfer of an alkyl group from *O*^6^-alkylguanine to N1 of a specific adenine in RNA. Two structures of this ribozyme were solved by X-ray crystallography, each as product complexes.^13,14^ The secondary structure of MTR1 can be described as a kind of three-way helical junction (Figure 1A). Within the core of the structure, the exogenous guanine product is coplanar with *N*^1^-methyladenine 63, C10, and U45, and held by a total of seven hydrogen bonds to the three nucleobases (Figure 1B).^13,14^ It is also stacked on both sides. Inspection of the structure indicated that if *O*^6^-methylguanine were bound in the same manner, it would be almost perfectly aligned for nucleophilic attack by A63 N1. Thus, the reaction would be accelerated by the effective local concentration of the *O*^6^-methylguanine, and the alignment of the reactants. However, the structure of the product-bound ribozyme coupled with mutagenesis suggested that there was another important factor contributing to catalysis. A C10U mutant exhibited undetectable alkyl transferase activity,^13^ and we therefore proposed the catalytic mechanism shown in Figure 1C in which the protonated form of C10 acts as a general acid to protonate N1 of *O*^6^-methylguanine. Analysis of the reaction trajectory by quantum mechanical calculation suggests that rather than occurring simultaneously, proton transfer from C10 precedes transfer of the alkyl group.^15^

Questions remain on the proposed mechanism, that we have probed using experimental and computational methods. First, can we strengthen the experimental evidence for the mechanism involving general acid catalysis by C10? Second, can we explore reactivity of the nucleophile A63? In this paper we have investigated the role of both the general acid and the nucleophile by means of atomic mutagenesis. The experimental data obtained are supported by quantum mechanical free energy simulations and are fully consistent with the proposed two-step mechanism.

## Experimental Section

### Synthesis of RNA Oligonucleotides

All oligonucleotides were synthesized using *t*-BDMS phosphoramidite chemistry.^16^ Unmodified oligonucleotides and that having an A(n7c) modification were synthesized on an Applied Biosystems 394DNA/RNA synthesizer as described in Wilson et al.,^17^ using UltraMILD ribonucleotide phosphoramidites^18,19^ (Link Technologies), and deprotected as described in Deng et al.^13^ The A(n7c) phosphoramidite was purchased from ChemGenes.

In this work we define atomic variants using lower case for the elemental name, with (original position new). For example, C10(n1c) is the cytosine at position 10 in the sequence with nitrogen at position 1 in the ring changed to carbon. The modified cytidine analogue amidites (Supplementary Figure S1) were prepared according to the procedure reported by Lu et al.^20^ and the RNA oligos containing C10(n1c) or C10(n1c, c5n) were synthesized with an Expedite 8909 synthesizer or H&A H-6 synthesizer in a standard 1 μmol scale protocol, followed by the standard deprotection and desilylation procedures.^21^

The MTR1 ribozyme was assembled from two RNA strands (Figure 1A):

MTR1 ribozyme (52 nt)

GCGGGCUGACCGACCCCCCGAGUUCGCUCGGGGACAACUAGACAUACAGUAU

MTR1 target (13 nt)

AUACUGAGCCCGC

The sites of incorporation of C(n1C) or C(n1c, C5n) into the ribozyme strand (C10) and A(n7c) into the target strand (A63) are underlined.

### Kinetic Analysis of MTR1 Ribozyme Catalyzed Alkyl Transfer

Standard single-turnover assays were performed at 37 °C in 25 mM MES (pH 6.0), 100 mM KCl, 40 mM MgCl_2_ and 50 μM *O*^6^-(4-aminomethyl-benzyl)-guanine, with 1 μM MTR1 ribozyme annealed to ∼20 pM radioactively-[5′-^32^P]-labeled target RNA. The RNA was annealed by rapid cooling from 80 °C in 50 mM KCl, to which buffer and salts were added followed by equilibration at 37 °C for 20 min. The reaction was initiated by addition of 200 μM *O*^6^-(4-aminomethyl-benzyl)-guanine that was also equilibrated to 37 °C. To investigate the pH dependence the buffers used were MES (pH 5.5–6.5), MOPS (pH 7.0–7.5) and TAPS (pH 8.0–8.5). We note that p*K*_a_ of the buffers used have a small temperature dependence; pH values were measured at room temperature, so there is an error of ∼0.15 pH units in kinetic measurements at 37 °C. Reactions were incubated under mineral oil to prevent evaporation and 2 μL aliquots were taken at time intervals and added to 13 μL 90% formamide, 50 mM EDTA to stop the reaction.

Unmodified and modified target RNA were separated by denaturing polyacrylamide gel electrophoresis using 14.4% gels (13.3% acrylamide, 1.1% bis-acrylamide) with 1× TBE and 7 M urea and band intensities were quantified by phosphorimaging. For both unmodified and variant ribozymes, the faster reactions exhibited biphasic kinetics with the reaction progress well fitted by a double exponential function:
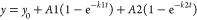
1

We assume that the faster rate reflects the rate of alkyl transfer and is reported as such, and that the slower rate reflects the conversion of a fraction of ribozyme initially folded in an inactive conformation. Slower reactions, typically less than 0.01 min^–1^, were well fitted by a single exponential presumably due to the rate of interconversion being faster than the rate of alkyl transfer. The rate constants for the fast and slow components are plotted in Supplementary Figure S2 and the fractions of each component are tabulated in Supplementary Table S1. Rate constants and uncertainties reported are the mean and standard deviation of at least three independent experiments. The apparent p*K*_a_ of the general acid (A) was evaluated using a single p*K*_a_ model for the observed rate:
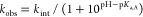
2where *k*_obs_ and *k*_int_ are the observed and intrinsic rate constants of the reaction, respectively.

### Computational Analysis of MTR1 Ribozyme Catalyzed Alkyl Transfer

All simulations were performed with AMBER22^22,23^ with molecular mechanical (MM) force field simulations utilizing GPU-accelerated MD.^24^ Combined quantum mechanical/molecular mechanical (QM/MM) simulations were performed with the sander program.^24^ A full description of the computational methods is given the Supporting Information.

Classical molecular dynamics (MD) simulations were based upon the unmodified MTR1 ribozyme crystal structure^13^ (PDB ID 7 V9E) containing *O*^6^-methylguanine (*O*^6^mG). C10 was protonated at the N3 position to enable its active state, established in previous studies.^15^ For simulations with the *O*^6^-(4-aminomethyl-benzyl) guanine (ab^6^G) ligand, the new group was modeled using the ligand structure in PDB ID 7Q7Z.^14^ The terminal amine of ab^6^G was protonated to have a +1 charge given the p*K*_a_ of benzylamine is ∼9.3.^25^ The system was described by the ff99OL3 force field,^26,27^ solvated in TIP4P-Ew^28^ water along with neutralizing Na^+^ and bulk concentration of 0.14 M NaCl using the Joung–Cheatham monovalent ion parameters.^29^ Electrostatics were treated using the particle mesh Ewald method.^30,31^ The simulation box was equilibrated in a stepwise manner as reported previously,^15^ 500 ns of aggregate production molecular dynamics (MD) simulations were performed for the unmodified system with each ligand. The final “active state” structure was used as a departure point for the three variants containing C10(n1c), C10(n1c, c5n), and A63(n7c). The modeled active state for all the systems had a proton at position 10 N3. The C10(n1c, c5n) also has a proton at N5 with a total +1 charge analogous to protonated cytosine, whereas C10(n1c) is neutral. The modified systems were shortly equilibrated and further simulated for 50 ns of production at constant pressure (*NPT*) at 298 K and 1 atm. A detailed analysis of the classical MD simulations can be found in the Supporting Information, including hydrogen bond analysis (Supplementary Figure S3), root-mean-square deviations (Supplementary Figure S4), and representative structures depicting interactions made by nucleotide 10 with the phosphate of A9 (Supplementary Figure S5).

Free energy surfaces and minimum free energy pathways for the chemical steps of the reaction were studied using a combined quantum mechanical/molecular mechanical model enhanced by a machine learning potential correction (QM/MM+ΔMLP). Specifically, the QM model was chosen to be DFTB3/3ob,^32,33^ a fast approximate density-functional tight-binding model for which extensive sampling could be achieved. A machine-learning potential was then trained to accurately correct the DFTB3/3ob model to a higher level ab initio density-functional PBE0/6-31G* model. The machine learning potential is the deep potential range corrected (DPRc) model^34,35^ implemented in DeePMD-kit.^36^ The resulting generalized hybrid model is designated DFTB3/3ob+ΔMLP_PBE0_, and has accuracy nearly indistinguishable from PBE0/6-31G* for the systems studied in the current work, but for computational cost reduced over 500-fold. Details regarding the machine learning training procedure and validation tests can be found in Supporting Information, including analysis of the training errors and their convergence shown in Supplementary Figures S6 and S7.

DFTB3/3ob+ΔMLP_PBE0_ umbrella sampling of the unmodified MTR1, C10(n1c), C10(n1c, c5n), and A63(n7c) variants with both ligands were performed to characterize the minimum free energy path. The QM region includes the nucleobases of nucleotides 10, 63, and the ligand. The unmodified MTR1, C10(n1c,c5n), and C10(n1c) QM regions with *O*^6^mG contained 46 atoms, whereas the A63(n7c) variant contains 47 atoms. These contained 62 and 63 atoms with ab^6^G. The QM region with *O*^6^mG of the unmodified MTR1, C10(n1c,c5n), and A63 (n7c) had a net charge of +1, whereas the C10(n1c) variant QM region was uncharged. With *O*^6^-4-aminomethyl-benzyl guanine, the net charges were increased by +1 in each case.

The reaction space was explored along coordinates that describe the proton transfer (PT), ξ_PT_ = *R*_C10:N3–H_ – *R*_*O*^6^alkG:N1–H_, and 4-aminomethyl-benzyl transfer (AT), ξ_AT_ = *R*_*O*^6^alkG: O6–CR_ – *R*_A63:N1–CR_ where *C*_R_ represents the electrophilic carbon with its respective R group. A linear initial guess at the minimum free energy path was made that connects the approximate position of the reactant (−1.5, −2.5 Å) and product (1.5, 2.5 Å) states. The path was discretized with 32 images. The minimum free energy path was obtained from 30 iterations of the surface accelerated string method (SASM).^37^ Each iteration of the string method sampled the images for 1 ps. Details regarding the DFTB3/3ob+ΔMLP_PBE0_ simulations and analysis can be found in Supporting Information, including 2D free energy surfaces (Supplementary Figure S8), analysis of correlated hydrogen bonding and partial charges (Supplementary Figure S9), comparison of free energy profiles with different alkyl donors (Supplementary Figures S10 and S11, and results for free energy profiles and representative structures for alternative mechanisms (Supplementary Figures S12–S14).

The p*K*_a_ shifts relative to the solution p*K*_a_ values were calculated for unmodified MTR1 and the three variants using alchemical free energy (AFE) simulations^24^ following our ProFESSA free energy workflow.^35^ Given that the experimental p*K*_a_ of C10 was effectively unperturbed between *O*^6^mG and ab^6^G, simulations were performed with *O*^6^mG. The end-states λ = 0 and λ = 1 (position 10:N3 protonated and not-protonated, respectively) were transformed over 25 λ windows using smoothstep softcore potentials^38^ applying the alchemical enhanced sampling (ACES)^39^ method. Free energies were calculated from the MBAR method^40^ implemented in FE-ToolKit.^41^ Details regarding the p*K*_a_ shift calculations can be found in the Supporting Information, including analysis of the calculated pH dependence of the rate of alkyl transfer by MTR1 as a function of the p*K*_a_ of the general acid and nucleophile (Supplementary Figure S15), and comparison of the computationally constructed activity-pH profile curves obtained by single versus double p*K*_a_ models (Supplementary Figure S16).

## Results

### Atomic Mutagenesis to Probe General Acid Catalysis by C10 in MTR1

**Figure 2:**
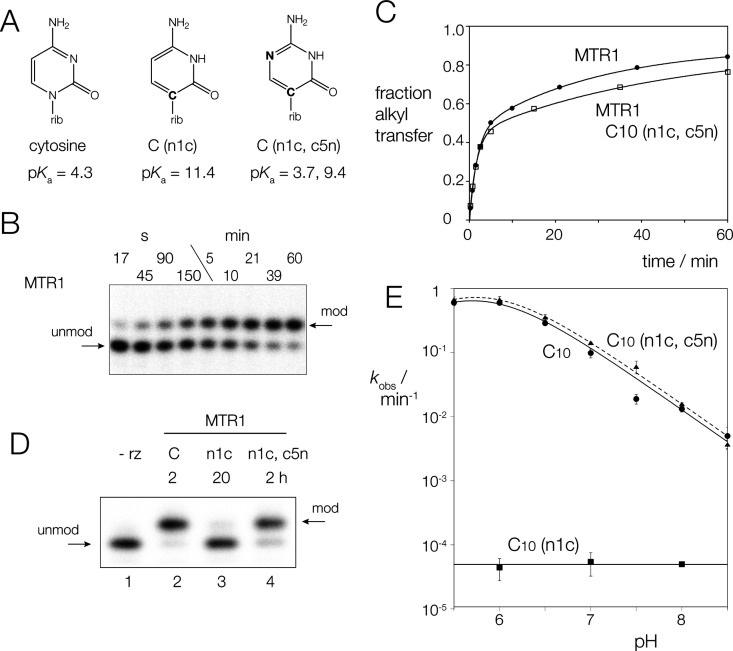
Investigation of the role of the proposed general acid C10 in alkyl transfer catalyzed by MTR1 using atomic variants of the cytosine. (A) Cytosine and it is atomic variants used in these studies. The p*K*_a_ values reported for the isolated nucleotides are shown. (B) A time course of alkyl transfer for the unmodified MTR1 ribozyme under our standard reaction conditions of 25 mM MES (pH 6.0), 100 mM KCl, 40 mM MgCl_2_ and 50 μM *O*^6^-(4-aminomethyl-benzyl)-guanine, 37 °C. The reaction times are shown above each lane. The unreacted RNA (unmod) migrates faster than the RNA modified by 4-aminomethyl-benzyl transfer (mod) in a polyacrylamide gel under denaturing conditions due to the acquisition by the modified RNA of the additional mass and a positive charge. (C) Reaction progress plotted as a function of time for the unmodified MTR1 ribozyme and C10-modified variants under standard reaction conditions. Data for MTR1 (filled circles; the reaction shown in part B) and C10(n1c, c5n)-containing MTR1 (open squares; the reaction shown in Supplementary Figure S2A) are plotted and fitted to two exponential functions (lines). (D) Gel showing 4-aminomethyl-benzyl transfer reactions for MTR1 and atomic variants under standard reaction conditions. Tracks: 1, substrate in the absence of MTR1 ribozyme; 2, unmodified MTR1 incubated with substrate for 2 h; 3, C10(n1c)-containing MTR1 incubated with substrate for 20 h; 4, C10(n1c, c5n)-containing MTR1 incubated with substrate for 2 h. (E) Observed rate constants (*k*_obs_/min^–1^) of 4-aminomethyl-benzyl transfer reactions for MTR1 and atomic variants in 25 mM buffer, 100 mM KCl, 40 mM MgCl_2_ and 50 μM *O*^6^-(4-aminomethyl-benzyl)-guanine at 37 °C plotted on a logarithmic scale as a function of pH. Error bars show standard deviations from ≥3 measurements. Filled circles, unmodified MTR1, filled squares MTR1 C10(n1c) and filled triangles C10(n1c, n5c). The data have been fitted to two ionizations (lines); the fit for C10(n1c, n5c) is shown as a broken line.

Previous studies of general acid–base catalysis in the nucleolytic ribozymes have used a combination of atomic mutagenesis and analysis of the dependence of kinetic rates upon pH^42−48^ as a powerful probe of chemical mechanism. We therefore sought atomic mutants of cytosine 10 that would perturb the p*K*_a_ of cytosine N3, that is the proton donor in our proposed catalytic mechanism (Figure 1C). Ideally such variants would not alter the chemical features of the Watson–Crick edge of the nucleobase, that interacts with the *O*^6^-alkylguanine in the ribozyme. A series of *C*-nucleoside variants of cytosine have been previously synthesized,^20,49^ of which two were considered promising for the analysis of the chemistry of MTR1 (Figure 2A).

(A) 1-Deazacytosine [C(n1c)] differs from cytosine in that the glycosidic N1 is replaced by carbon, resulting in a very high p*K*_a_ of 11.4 for the isolated nucleotide. The Watson–Crick edge of the nucleobase is unchanged, but N3 should no longer be effective as a general acid to protonate N1 of *O*^6^-alkylguanine.

(B) Pseudo isocytosine [C(n1c, c5n)] can be considered as a further modification of C10 (n1c) where C5 is replaced by nitrogen. Once again the Watson–Crick edge of the nucleobase is unchanged, but the isolated nucleotide now has p*K*_a_ values of 3.7 and 9.4, so potentially restoring a low p*K*_a_ value.

Variant MTR1 ribozymes were synthesized as two RNA oligonucleotides in which C10 in the ribozyme strand was replaced by either C10(n1c) or C10(n1c, c5n). While the p*K*_a_ values of the modified nucleotides are likely to be altered by the electrostatic environment of the folded RNA,^50^ the basic trends should remain similar. These p*K*_a_ shifts in the MTR1 environment have been calculated from free energy simulations described below.

### Rates of Alkyl Transfer of Atomic Mutants of C10 in MTR1

In these experiments the rate of alkyl transfer catalyzed by MTR1 was measured by gel electrophoresis of substrate and product RNA as a function of time using *O*^6^-(4-aminomethyl-benzyl)-guanine (Figure 1D). The migration of the 13 nt substrate RNA becomes significantly retarded when the target A63 is converted to *N*^1^-(4-aminomethyl-benzyl)-adenine due to a combination of the positive charge acquired, and the additional mass of the 4-aminomethyl-benzyl group (Figure 2B). The rate constant 4-aminomethyl-benzyl transfer by the unmodified ribozyme at pH 6.0 is *k*_obs_ = 0.60 ± 0.01 min^–1^ (Figure 2C). The alkyl transfer activity of the two atomic variants has been analyzed under the same conditions (Figure 2D) and all rates tabulated in Table 1.

**Table 1:** Measured Rate Constants (/min^–1^) for 4-Aminomethyl-benzyl Transfer for MTR1 and Atomic Variants at the Indicated pH Values

	pH 6.0	SD	rel. rate	pH 7.5	SD	rel. rate	pH 8.0	SD	rel. rate
C10/A63	0.6	0.01	–1	0.019	0.003	0.032	0.013	0.001	0.022
C10 n1c	4.4 × 10^–5^	1.7 × 10^–5^	7.3 × 10^–5^				5.0 × 10^–5^	4.0 × 10^–6^	8.3 × 10^–5^
C10 n1c c5n	0.67	0.08	1.1	0.06	0.01	0.1	0.0158	0.0006	0.026
A63 n7c	5.1	0.4	8.5	0.52	0.03	0.87	0.38	0.06	0.63

Upon replacement of C10 N1 by carbon (C10(n1c)), 4-aminomethyl-benzyl transfer became very slow (Figure 2D (track 3)), with a very small extent of alkyl transfer occurring in 20 h. Thus, raising the p*K*_a_ to a nominal value of 11.4 led to a marked loss of catalytic activity. However, on making a second, additional substitution of C10 C5 by nitrogen C10(n1c, c5n) alkyl transfer was restored (Figure 2D (track 4) and Supplementary Figure S17), with a rate of 0.67 ± 0.08 min^–1^ at pH 6.0 (Figure 2C). Thus, making a second substitution that provides a lower p*K*_a_ value completely restored the rate of 4-aminomethyl-benzyl transfer despite retention of the first n1c substitution.

We have compared the effect of pH on the rate constant for 4-aminomethyl-benzyl transfer for the unmodified and the C10 variant-containing ribozymes (Figure 2E). For the unmodified MTR1 ribozyme the rate constant for alkyl transfer decreases above pH 6.0, corresponding to an apparent p*K*_a_ value of 6.4 ± 0.1. This is very similar to the pH dependence of methyl transfer that we have measured previously.^13^ The pH profile for the C10(n1c, c5n)-containing ribozyme is closely similar, corresponding to an apparent p*K*_a_ value of 6.5 ± 0.1. Thus, the inclusion of the second substitution (c5n in addition to n1c) restored both the activity and pH dependence of the alkyl transfer reaction to be closely similar to the unmodified ribozyme.

Although we observed a major loss of activity for the MTR1 C10(n1c) variant, the ribozyme was not totally inactive. Allowing the reaction to proceed for 6 days led to a measurable extent of alkyl transfer (Supplementary Figure 17B,C), from which we calculated a rate constant of *k*_obs_ = (4 ± 2) × 10^–5^ min^–1^. The rate was measured as a function of pH (Figure 2E), revealing that the rate of alkyl transfer was independent of pH over the observed range.

### Computational Analysis of the Role of C10 in General Acid Catalysis

**Figure 3:**
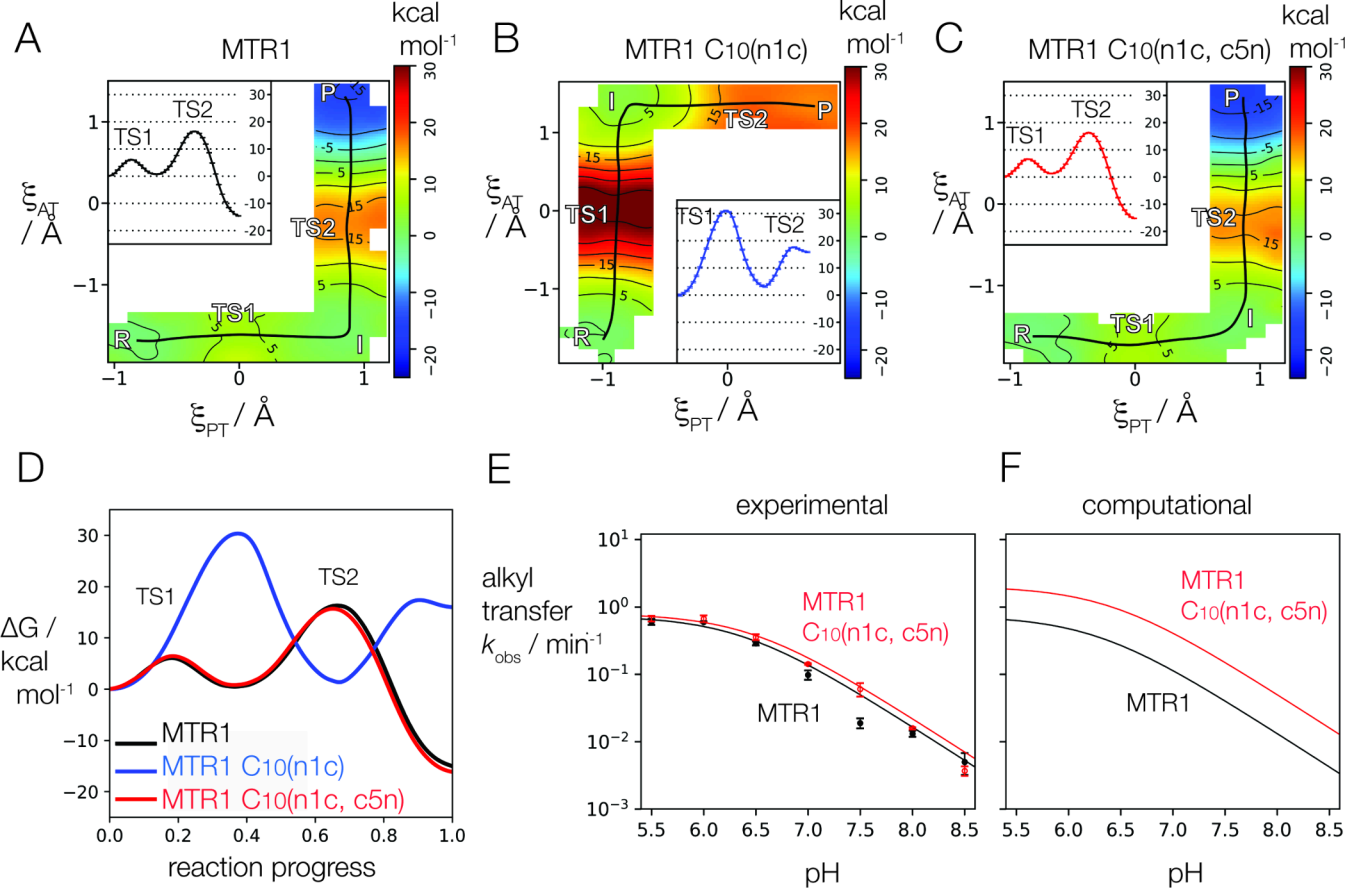
Computational analysis of the role of C10 as general acid in catalysis by MTR1. (A) Free energy surface around the minimum free energy path and corresponding free energy profile of the MTR1 reaction with *O*^6^-(4-aminomethyl-benzyl) guanine as an alkyl donor at the DFTB3/3ob+ΔMLP_PBE0_ level of theory. ξ_AT_ and ξ_PT_ (here and in parts B and C) are the coordinates of alkyl and proton transfer respectively as defined in Materials and Methods. Inset shows the free energy profile (black) along the minimum free energy path. (B) Free energy surface around the minimum free energy path and corresponding free energy profile of the C10(n1c) reaction with *O*^6^-(4-aminomethyl-benzyl) guanine as an alkyl donor at the DFTB3/3ob+ΔMLP_PBE0_ level of theory. Inset shows the free energy profile (blue) along the minimum free energy path. (C) Free energy surface around the minimum free energy path and corresponding free energy profile of the C10(n1c,c5n) reaction with *O*^6^-(4-aminomethyl-benzyl) guanine as an alkyl donor at the DFTB3/3ob+ΔMLP_PBE0_ level of theory. Inset shows the free energy profile (red) along the minimum free energy path. (D) Comparison of the free energy profiles along the minimum free energy path for MTR1 (black), C10(n1c) (blue), and C10(n1c,c5n) (red). (E) Experimental pH dependence of the rate of 4-aminomethyl-benzyl transfer by MTR1 and C10(n1c, c5n)-containing MTR1. (F) Computed pH dependence of the rate of alkyl transfer by unmodified MTR1 and C10(n1c, c5n)-containing MTR1. The computationally observed rates are normalized such that the maximum of the MTR1 curve (black) is aligned to maximum of the experimental fit.

Combined QM/MM simulations together with alchemical free energy simulations were used to compute the activity-pH profiles analogous to the experimentally measured values for MTR1, C10(n1c), and C10(n1c, c5n) (Figure 3). System setup and additional analysis are provided in Supporting Information (Supplementary Figures S3–S5). The profiles require calculation of the intrinsic rate defined in eqs S1–S3 of the Supporting Information, as derived from the minimum free energy path for the reaction (Figure 3A–C), as well as the p*K*_a_ shift of the N3 position of the nucleotide at position 10. The calculated intrinsic rates and p*K*_a_ shifts are listed in Table 2 and the free energy profiles are compared in Figure 3D. The p*K*_a_ of C10(n1c) in the ribozyme environment is predicted to be raised by approximately 8–10 units with respect to that of C10, strongly disfavoring proton transfer in the initial step. Under the mechanistic assumption that proton transfer can only occur from the N3 position of C10(n1c), computations predict a very high activation free energy and a rate that is below the experimental detection limit (Figure 3B,D). Experimentally, there appears a very slow residual background rate, just within the detection limit, that is pH-independent and likely arises from an alternative mechanism. Although the p*K*_a_ in aqueous solution (p*K*_a_ = 3.7) of cytosine (n1c, c5n) is slightly lower than for cytosine (p*K*_a_ = 4.2), the additional endocyclic nitrogen forms stabilizing hydrogen bonds in the simulation that causes a greater increase in p*K*_a_ in the ribozyme environment relative to cytosine (see Supplementary Figure S5). This leads to predicted p*K*_a_ values for the N3 position that are very similar in MTR1 and the C10(n1c, c5n) variant (p*K*_a_ = 6.3 and 6.6, respectively), and in quantitative agreement with the experimental apparent p*K*_a_ values (p*K*_a_ = 6.4 ± 0.1 and 6.5 ± 0.1, respectively). The rate-controlling transition state corresponds to the alkyl transfer step and is 0.6 kcal mol^–1^ lower in barrier than the unmodified ribozyme, leading to similar free energy profiles and intrinsic rates (Table 2). Thus, the calculated trends are in close agreement with experiment given the error range (Figure 3E,F), and support a mechanism for alkyl transfer facilitated by general acid catalysis by C10. Additional details of the free energy profiles including error estimates are given in Supplementary Tables S2 and S3. The pathways corresponding to two possible stepwise mechanisms (proton transfer preceding methyl transfer, and vice versa) are compared on the computed full 2-dimensional free energy surfaces shown in Supplementary Figure S8.

**Table 2:** Summary of Thermodynamic and Estimated Kinetic Quantities for the Reactions of MTR1 Variants from DFTB3/3ob+ΔMLP_PBE0_ and Alchemical Free Energy Simulations in Comparison to Experimental Values

	kcal·mol^–1^	calc^a^	expt	expt, soln^b^	soln → MTR1	MTR1^c^	expt, MTR1
species	Δ*G*^‡^	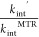	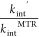	p*K*_a_	Δp*K*_a_	p*K*_a_	p*K*_a,apparent_
MTR1	16.3	1.0	1.0	4.2	2.1	6.3	6.4
C_10_ (n1c)	30.3	∼10^–11^	∼10^–4^	11.4	5.2	16.6	d
C_10_ (n1c, c5n)	15.7	2.7	1.1	3.7	2.9	6.6	6.5
A_63_ (n7c)	15.4	4.3	6.1	4.2	2.1	6.3	6.9

a *k*_int_^′^ refers to the intrinsic rate constant of an MTR1 atomic variant.

b The value refers to the experimental p*K*_a_ of the lone nucleobase in solution for the nucleotide at position 10 of the ribozyme.

c p*K*_a_ values are calculated for nucleotide 10 of the ribozyme.

d No pH dependence observed.

### Analysis of the Reactivity of the A63 Nucleophile on the Rate of Alkyl Transfer

**Figure 4:**
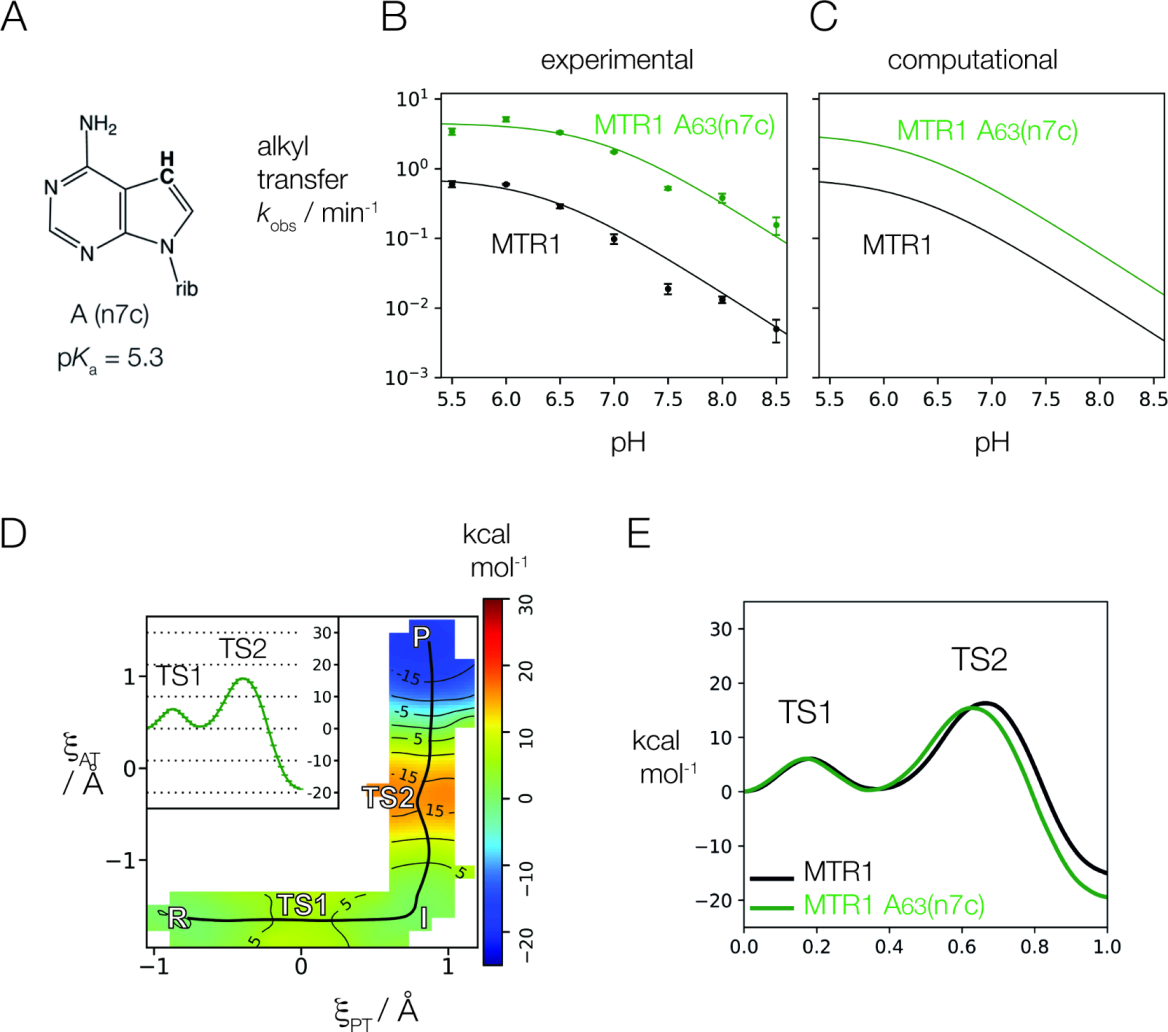
Analysis of the reactivity of A63 as the nucleophile in alkyl transfer by MTR1. (A) The structure of 7-deazaadenine (A(n7c)). This has a p*K*_a_ value of 5.3, raised from the value of adenine of 3.5. (B) The effect of A63(n7c) on the observed rate of 4-aminomethyl-benzyl transfer catalyzed by MTR1 as a function of pH, compared to unmodified MTR1. The green filled circles are the data obtained from MTR1 A63(n7c); error bars show standard deviations on ≥3 measurements. The black circles are the data from unmodified MTR1, and are the same data as shown in Figure 2E. The observed rate of alkyl transfer is 6-fold higher for the MTR1 A63(n7c) variant uniformly over the whole pH range. (C) The computed rates of alkyl transfer for unmodified MTR1 (black) and MTR1 A63(n7c) (green). Note that the calculated rates reproduce a similar increase in rate due to the A63(n7c) substitution. (D) Calculated potential energy surface for alkyl transfer catalyzed by the MTR1 A63(n7c) variant. ξ_AT_ and ξ_PT_ are the coordinates of alkyl and proton transfer respectively as defined in Materials and Methods. (E) The calculated free energy along the reaction coordinate computed for MTR1 unmodified (black) and A63(n7c) (green).

We have studied the rate of alkyl transfer by MTR1 in which the nucleophile in the reaction (A63) was modified by an N7 deaza-substitution i.e., A63(n7c) (Figure 4A). This raises the p*K*_a_ of the free nucleotide to 5.3 from 3.7 in adenine. The effect of this atomic substitution on the rate of alkyl transfer by MTR1 is a 6-fold increase in rate constant uniformly over the complete range of pH from 5.5 to 8.5 (Figure 4B and Table 1). These data indicate an elevated reactivity of the A63 nucleophile, consistent with a significant extent of C–N bond formation in the transition state. We discuss this further below in the Discussion.

Figure 4D,E shows the calculated 2D free energy surface for the A63(n7c) variant, and compares the 1D free energy profile with that of the unmodified MTR1. The calculations predict that A63(n7c) has a slightly lower rate-controlling TS barrier, leading to a slightly increased intrinsic rate relative to the unmodified MTR1 (Figure 4B and Table 2). The predicted relative intrinsic rate 4.3 is in good agreement with the experimentally measured value of 6.1. While the N7 position of A63 does not participate in any hydrogen bonds within the RNA, we found that the hydrogen bond between the exocyclic amine of A63 and the N7 of the ligand was generally shorter upon n7c substitution. Analysis of the accumulated electronic charge of the exocyclic nitrogen (shown in Supplementary Figure S9) showed that increasing the nucleophilicity of N1 draws negative charge away from the amine, making it a better hydrogen bond donor.

### Analysis of the pH Dependence of Alkyl Transfer at Low pH

The A63(n7c) substitution is informative from a second point of view. We^13^ and Höbartner and co-workers^14^ observed that at pH values below 5.5 the rate of alkyl transfer decreased with reduction in pH. In principle this might result from protonation of A63 N1, which would render it unable to accept alkyl transfer from the *O*^6^-alkylguanine. However, if this were correct we would expect to observe a shift in the downturn into the measurable range with the higher p*K*_a_ of A63(n7c) if the solution p*K*_a_ shift between the two nucleotides holds in the ribozyme environment. This is illustrated by the calculated pH profiles shown in Supplementary Figure S15, showing that if the downturn were due to protonation of A63, then substitution by A63(n7c) would lead to a readily detectable shift in the maximal rate with pH under this assumed shift. In fact the shape of the pH profile was altered very little by the A63(n7c), with a calculated p*K*_a_ value of 6.9 ± 0.2, with no pronounced reduction in rate at low pH over the range studied here (Figure 4B). This suggests that A63 is unlikely to be responsible for the lowering of rate at low pH, and that the p*K*_a_ of A63 must be below the measurable range. Computational study of the p*K*_a_ of A63(n7c) shows a downshift from the solution value of 5.3 to 4.8 in the ribozyme, indicating a 2 p*K*_a_ unit difference between A63(n7c) and A63, which in previous computational work^15^ was found to downshift to 2.8. As shown in Supplementary Figure S16 of the Supporting Information, these p*K*_a_ values would be barely perceptible when fitting with a two p*K*_a_ model as opposed to a single p*K*_a_ model in the experimentally relevant pH range. Therefore, the suggestion of a downturn in rate due to A63 protonation at low pH is not mechanistically significant under the experimental conditions.

## Discussion

Crystal structures of the MTR1 ribozyme bound to its guanine product^13,14^ suggested a catalytic mechanism involving the nucleobase of C10 acting as a general acid to transfer a proton to N1 of *O*^6^-alkylguanine (Figure 1C). This was consistent with the substantial loss of activity by a C10U variant of the ribozyme, and the pH dependence of the alkyl transfer reaction.^13,14^ In the present study we have studied two atomic variants of C10 to probe the mechanism further. The variants do not perturb the key Watson–Crick edge of the cytosine, but markedly alter the p*K*_a_ values. The first variant (changing N1 to C, and thus raising the p*K*_a_ substantially) led to a very slow rate of reaction. Addition of a second modification (changing C5 to N, thereby generating a new p*K*_a_ close to 3.7 as the isolated nucleotide) led to a complete restoration of activity. Thus, the first change results in around a ∼10^4^-fold loss of activity, and then adding the second modification restores normal alkyl transfer activity. This is hard to explain in terms other than the proposed mechanism, and provides a powerful confirmation of the mechanism involving general acid catalysis by C10. The experimental results are furthermore in excellent agreement with the computational studies, discussed further below.

Our previous computational analysis suggested a two-step consecutive mechanism in which the proton transfer from C10 precedes alkyl transfer to A63 N1,^15^ and the large activation barrier for the alkyl transfer step has been confirmed in the present analysis. This is supported by the new results from the A63(n7c) substitution. Alkyl transfer by the MTR1 ribozyme carrying this modification is 6-fold faster than for the unmodified ribozyme, indicating that the reactivity of the nucleophile has increased. The deaza modification at adenine N7 raises its p*K*_a_ by 1.5 units (changing from 3.7 to 5.3 in the isolated nucleotides), and application of the Bro̷nsted equation^51^
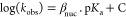
gives a β_nuc_ for the reaction of 0.5. This indicates significant bond formation between the adenine N1 nucleophile and the C of the alkyl group in the transition state. This is consistent with alkyl transfer being rate limiting as indicated by QM calculations.

Computational analysis supports the mechanism for the unmodified ribozyme in which facile proton transfer from C10 followed by alkyl transfer in the rate controlling step. The rate controlling TS2 is an “early” transition state in the sense that the reaction coordinate value ξ_AT_ of −0.24 Å (see Supplementary Table S3) corresponding to nucleophilic attack that is less advanced (N1–C6 distance 2.26 Å) than that of the leaving group departure (C6–O6 distance 2.02 Å). Relative to the rate controlling TS2, the proton transfer can be considered as in a rapid equilibrium between C10 and *O*^6^alkylguanine with significant population in both states (R and I in Figure 3A). The C10(n1c) variant introduces a large p*K*_a_ shift (7.2 units) at the N3 position that will be further increased by the ribozyme environment (Table 2) to a total of approximately 10 units. This theoretically prevents the initial proton transfer step and forces the reaction to proceed by an alternative high barrier path. This interpretation is strongly supported by examination of the C10(n1c, c5n) substitution that completely rescues the activity from that of the 1 C10(n1c) variant by introducing a second endocyclic amine modification that brings back the p*K*_a_ of the general acid to be nearly identical to that of the unmodified MTR1 (Table 2). Examination of the A63(n7c) substitution does not affect the general acid, but rather increases the nucleophilicity of the N1 position of A63. This causes an increase in the intrinsic rate that results from reducing the free energy barrier of the methyl transfer step (TS2) and stabilizing the product (P). The increase in nucleophilicity is supported by noting the TS2 is an even “earlier” transition state with ξ_AT_ value of −0.25 Å. This is consistent with the interpretation from linear free energy relationships that suggests raising the free energy of the initial state of the reaction step will lower the barrier and shift the transition state toward the initial state.^52^

A consistent theme across all variants studied both experimentally and computationally is that the *O*^6^-(4-aminomethyl-benzyl) guanine reacts significantly faster than *O*^6^-methyl guanine. The electron withdrawing property of the benzylamine side chain would be expected to increase the electrophilicity of the reacting carbon atom. In addition, we hypothesize that the benzylamine side chain could enhance the stabilizing interaction between C10 and the guanine reactant. As shown in Supplementary Figures S10, S11, and Supplementary Table S2, we predict the free energy barrier for 4-aminomethyl-benzyl transfer to be 1.1 kcal mol^–1^ lower than that of methyl for the unmodified ribozyme, which translates to an approximately 47-fold increase in intrinsic rate. In the case of C10(n1c) modification, there is no positively charged intermediate, thus the barriers predicted with *O*^6^-methyl guanine and *O*^6^-(4-aminomethyl-benzyl) guanine of 30.8 and 30.3 kcal mol^–1^, principally reflects the strength of the electrophile. Given that this reaction is experimentally detected, some other process must act to lower the free energy barrier.

The origin of the small residual activity of MTR1 C10(n1c) is not fully understood at this time. The remaining activity is 10^4^-fold reduced compared to the unmodified ribozyme, and independent of pH. This might reflect the effects of approximation and orientation, but the rate is probably too high for this alone. Clearly C10(n1c) is no longer effective as a general acid. It is possible another element in the RNA could donate a proton to the *O*^6^-alkylguanine. We have explored various plausible alternative mechanisms that involve proton transfer from U45, the exocyclic amine of C10(n1c), and the exocyclic amine of A63 (see Supporting Information). Proton transfer from U45 was predicted to be the most favorable mechanism based on the free energy barriers. We also investigated the potential effect of metal ion binding to promote alternative mechanisms. Details can be found in Supplementary Figures S12–S14 and Supplementary Table S4 of the Supporting Information.

**Figure 5:**
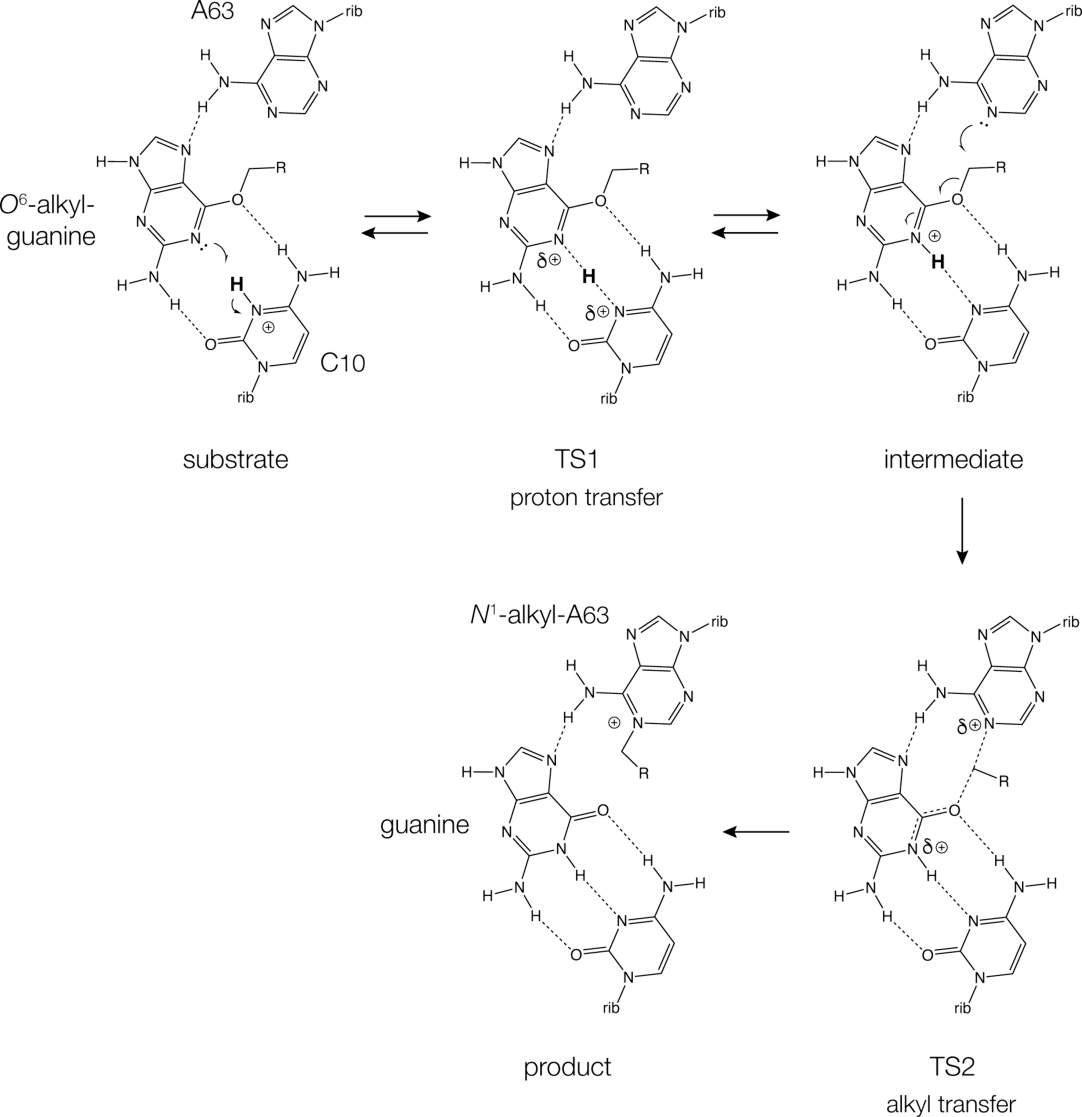
Proposed two-step reaction mechanism for alkyl transfer my MTR1 proposed on the basis of these studies. There is a relatively facile transfer of a proton between C10 N3 and *O*^6^-alkylguanine N1, proceeding via the transition state TS1. Subsequently, alkyl transfer proceeds only from the state in which *O*^6^-alkylguanine N1 is protonated, via transition state TS2 to give the product *N*^1^-alkyladenine, and unprotonated C10.

In conclusion, the alkyl transferase ribozyme MTR1 catalyzes the transfer of an alkyl group from *O*^6^-alkylguanine to N1 of the target adenine in two stages, shown in Figure 5. The reaction is subject to general acid catalysis by the nucleobase of cytosine C10. This mechanism is supported experimentally by the atomic variants studied here, and by quantum mechanical modeling of the reaction. The observed increase in reaction rate with raised p*K*_a_ of A63 indicates significant C–N bond formation in the transition state. This is consistent with the computational analysis indicating a two-stage reaction where the energy barrier for alkyl transfer is significantly larger than that for proton transfer from C10 N3 to *O*^6^-alkylguanine N1. The lower energy barrier for proton transfer indicates that the proton should shuttle back and forth between C10 and the *O*^6^-alkylguanine, but alkyl transfer will only occur from the form in which the proton resides on *O*^6^-alkylguanine N1. Protonation of *O*^6^-(4-aminomethyl-benzyl)-guanine will create a better electrophile for the alkyl transfer reaction. We now have a deeper understanding of the catalytic mechanism of this ribozyme, and furthermore the study has demonstrated the power of combining experimental and computational analysis.

We have sought mechanistic comparisons with alkyl transfer catalyzed by protein enzymes. The great majority of methyltransferase enzymes utilize *S*-adenosylmethionine (SAM) as the methyl donor; these do not require proton transfer as the methyl group is transferred from a positively charged sulfonium center. However, the demethylation of *O*^6^-methylguanine by the *O*^6^-alkylguanine-DNA alkyltransferase (AGT) protein has distinct mechanistic parallels. The structure of AGT has been determined,^53^ and the mechanism investigated computationally.^54−56^ These studies suggest that an active site cysteine is first deprotonated by a water-mediated histidine prior to methyl transfer, but the transfer leaves a negatively charged guanine nucleotide. The work of Georgieva and Himo^55^ suggests an additional step in the mechanism of the human AGT protein whereby a nearby tyrosine side chain transfers a proton to the N3 position of the negatively charged guanine, and subsequently a neighboring lysine residue transfers a proton to the tyrosine such that all species in the product state are neutrally charged. This is intriguing given that upon C10(n1c) substitution in MTR1, we predicted a plausible alternate mechanism involving proton transfer to the N3 of the guanine ligand. In the case of the AGT protein, the nucleophile is negatively charged, thus a proton transfer to *O*^6^-methylguanine preceding methyl transfer is not necessary. However, the mechanistic pathway is reversed in MTR1 as the nucleophile is neutrally charged and the electrophile must be activated.

Despite being the product of relatively few rounds of in vitro selection, MTR1 is a remarkably sophisticated ribozyme, employing a chemical mechanism involving nucleobase-mediated general acid catalysis. This provides proof of principle that ribozymes carrying out a wider range of catalytic chemistry could have been operating in an RNA world.

## Supporting Information Available

Supplementary Methods, Supplementary Results, 17 supplementary figures, and 4 supplementary tables (PDF)
