# Supplementary material for: The Role of General Acid Catalysis in the Mechanism of an Alkyl Transferase Ribozyme
Source: ACS Catal. 2024 Oct 2;14(20):15294–305. doi: 10.1021/acscatal.4c04571 (PMC11494507; doi:10.1021/acscatal.4c04571)
Supplement: Supplementary file 1 — cs4c04571_si_001.pdf [file cs4c04571_si_001.pdf]

The role of general acid catalysis in the mechanism of an alkyl transferase ribozyme

Timothy J. Wilson[1], Erika McCarthy[2], Şölen Ekesan[2], Timothy J. Giese[2], Nan-Sheng Li[3], Lin Huang[4], Joseph A. Piccirilli[3,5], Darrin M. York[2] and David M. J. Lilley[1]

1. Nucleic Acid Structure Research Group, Division of Molecular, Cellular and Developmental Biology, MSI/WTB Complex, The University of Dundee, Dow Street, Dundee DD1 5EH, U.K
2. Laboratory for Biomolecular Simulation Research, Institute for Quantitative Biomedicine and Department of Chemistry and Chemical Biology, Rutgers University, Piscataway, NJ 08854, United States
3. Department of Chemistry, The University of Chicago, Chicago, Illinois 60637, United States
4. Guangdong Provincial Key Laboratory of Malignant Tumor Epigenetics and Gene Regulation, Guangdong-Hong Kong Joint Laboratory for RNA Medicine, Sun Yat-sen Memorial Hospital, Sun Yat-sen University, Guangzhou 510120, P.R. China ; Medical Research Center, Sun Yat-sen Memorial Hospital, Sun Yat-sen University, Guangzhou 510120, P.R. China.
5. Department of Biochemistry and Molecular Biology, The University of Chicago, Chicago, Illinois 60637, United States

Correspondence should be addressed : Darrin.York@rutgers.edu, d.m.j.lilley@dundee.ac.uk

SUPPORTING INFORMATION

SUPPLEMENTARY METHODS

Computational methods

All molecular mechanical (MM) force field simulations were performed with the GPU-accelerated version of pmemd.[1-2] Combined quantum mechanical/molecular mechanical (QM/MM) simulations were performed with the sander program.[3] Unless otherwise stated, all molecular dynamics simulations were performed with a 1 fs time step. The SHAKE algorithm[4] was used to fix MM bonds involving hydrogen, whereas all QM bonds were left unconstrained. Temperatures were regulated with a Langevin thermostat[5] with a collision frequency of 5 ps^{-1}. Pressures were controlled with the Berendsen barostat.[6] The MM simulations calculated electrostatics with the particle mesh Ewald method[7-9] using tinfoil boundary conditions[10-11] a 1 $Å^3$ reciprocal space grid, and 10 Å real space cutoffs. The semiempirical QM/MM simulations similarly calculated long-range electrostatics with an Ewald method that uses Mulliken charges,[12-13] and *ab initio* QM/MM calculations used the ambient potential composite Ewald method.[14] The Lennard-Jones interactions were calculated to 10 Å and a long-range tail correction was included to account for the interactions beyond the cutoff.[15] Covalent bonds at the QM/MM boundary were capped with the hydrogen link-atom approach.[16-17] Alchemical free energy simulations were performed with the modified SSC(2) softcore potential and the one-step concerted softcore protocol.[18] The unitless parameters of the softcore potential are: m=2, n=2, α=0.5, and β = 1.

System preparation

The unmodified MTR1 ribozyme was prepared departing from the crystal structure (PDB ID 7V9E[19]) containing the cofactor O^6mG. The C10 nucleotide was protonated at the N3 position to represent the ribozyme active state.[19-21] For simulations with the ab^{6}G cofactor, the new group was modelled in by aligning the cofactor coordinates from PDB ID 7Q7Z containing ab^{6}G. The terminal amine of ab^{6}G was protonated to have a +1 charge given the pK_a of benzylamine is ~9.3.[22] The structure consists of 2,207 atoms (2223 with ab^{6}G) with a net 66− charge which was solvated with 18,250 TIP4P/Ew waters, 113 sodium ions, and 47 chlorine ions in a truncated octahedron with 90.2 Å real space lattice vectors resulting in 75,367 particles (75383 with ab^{6}G) and an ion concentration of 140 mM. The MM potential was modelled with the ff99OL3 RNA force field[23] and Joung and Cheatham[24] monovalent ion parameters. Details regarding the preparation and equilibration of this system have already been reported elsewhere.[20] In brief, the pressure and temperature were equilibrated for 50 ns with the MM force field potential to maintain 1 atm and 298 K in the isothermal-isobaric ensemble.

Three chemically modified variants of the ribozyme were prepared in a similar fashion. The variants are referred to as C10(n1c,c5n), C10(n1c), and A63(n7c). The C10(n1c,c5n) system replaces the N1 position with a carbon and the C5 position with a nitrogen. Furthermore, it protonates C10 at both the N3 and N5 positions, resulting in a net +1 charge on the modified C10 nucleotide. The C10(n1c) variant differs from unmodified MTR1 by replacing the N1 position with a carbon, resulting in a net neutral modified C10 nucleotide. The A63(n7c) variant differs from the unmodified MTR1 by replacing the N7 position with a C. The atomic charges of the modified nucleotides as well as the cofactor were determined from the restrained electrostatic potential

(RESP) charge fitting procedure,[25] where the potential was calculated at HF/6-31G*. Missing bonded parameters were extracted from the GAFF2 force field.[1] The 3 variant structures departed from the equilibrated unmodified MTR1 structure. After the modifications were made, the structure was minimized with 500 steps of the unrestrained conjugate gradient method. The system was heating from 0 K to 300 K over 1.6 ns at constant volume, and 50 ns of additional simulation was performed in the *NPT* ensemble at 298 K and 1 atm.

QM/MM umbrella sampling

QM/MM umbrella sampling of the unmodified MTR1, C10(n1c,c5n), C10(n1c), and A63(n7c) variants were performed to characterize the minimum free energy path. The sampling was performed multiple times with different QM Hamiltonians, including the DFTB3/3ob tight binding model (using the “3ob” parameter set),[26] *ab initio* PBE0/6-31G*, and a semiempirical model corrected with a machine learning potential, DFTB3/3ob+ΔMLP$_{\text{PBE0}}$, described in the next section. Ultimately, we seek to make a best estimate of the reaction profiles at the *ab initio* PBE0/6-31G* QM/MM level of theory; however, its high computational cost prohibits sampling on a long time scale. To circumvent this limitation, we develop a cost effective DFTB3/3ob+ΔMLP$_{\text{PBE0}}$ model that can be sufficiently sampled.

The QM region includes the nucleobases of nucleotides 10, 63, and the cofactor. The unmodified MTR1, C10(n1c,c5n), and C10(n1c) QM regions with O^6mG contained 46 atoms, whereas the A63(n7c) variant contains 47 atoms. These contained 62 and 63 atoms with ab^{6}G. The QM region with O^6mG of the unmodified MTR1, C10(n1c,c5n), and A63(n7c) had a net charge of +1, whereas the C10(n1c) variant QM region had a neutral charge. With ab^{6}G the net charges were +2 and +1. The reaction free energy was explored with umbrella sampling using 2 reaction coordinates that describe the proton transfer (PT), $\xi_{\text{PT}} = \text{R}_{\text{C10:N3-H}} - \text{R}_{O^6\text{alkG:N1-H}}$, and alkyl transfer (AT), $\xi_{\text{AT}} = \text{R}_{O^6\text{alkG:O6-C}_\text{R}} - \text{R}_{\text{A63:N1-C}_\text{R}}$ where C_R represents the electrophilic carbon with its respective R group. Umbrella sampling was performed with 100 kcal mol^{-1} Å^{-2} force constants. A linear initial guess at the minimum free energy path was made that connects the approximate position of the reactant (-1.5 Å, -2.5 Å) and product (1.5 Å, 2.5 Å) states. The path was discretized with 32 images. Initial coordinates for the images were prepared from a sequence of brief 500 fs simulations. These images were then equilibrated for 5 ps in the *NVT* ensemble at 298 K. The minimum free energy path was obtained from 30 iterations of the surface accelerated string method.[27] Each iteration of the string method sampled the images for 1 ps. The free energy surface was calculated from the aggregate sampling obtained from all iterations by solving the multistate Bennett acceptance ratio[28] (MBAR) and unbinned weighted histogram[29-30] (UWHAM) equations, as implemented in the FE-ToolKit software.[31] 4 trials of production simulations were performed along the minimum free energy path for 200 ps per trial. Each trial used one of the four stochastically trained MLP’s and was initiated from a different random number seed. The final free energy profiles presented are an average over the four trials with the associated bootstrap standard errors.

Additionally, a series of QM/MM simulations were conducted to probe potential mechanisms of the C10(n1c) system where the alkyl transfer coordinate was maintained, and the proton transfer coordinates tested were: $\xi_{\text{PT}} = \text{R}_{\text{C10:N4-H}} - \text{R}_{O^6\text{alkG:O6-H}}$, $\xi_{\text{PT}} = \text{R}_{\text{A63:N6-H}} - \text{R}_{O^6\text{alkG:N7-H}}$, and $\xi_{\text{PT}} = \text{R}_{\text{U45:N3-H}} - \text{R}_{O^6\text{alkG:N3-H}}$. In the case of proton transfer from U45, this nucleotide was included

in the QM region and C10 was treated with the MM forcefield (60 atoms, net neutral charge). A case with Mg^{2+} coordinated to U45:O4 included Mg^{2+}, 4 coordinating waters, and the phosphate of G41 in the QM region (78 atoms, net +2 charge). Each mechanism was initially screened at the DFTB3/3ob level using between 20 and 30 string iterations, and PBE0 level production simulations were conducted for 5 ps for the promising U45 proton transfer mechanisms.

Training of the machine learning potential correction

The DFTB3/3ob+ΔMLP_{PBE0} model uses the so-called ΔMLP_{PBE0} approach,[32] which supplements an inexpensive semiempirical Hamiltonian with nonelectronic neural network corrections that are parametrized to reproduce high-level, target energies and forces. In the present work, the semiempirical model is DFTB3/3ob, the target energies and forces are calculated from PBE0/6-31G*, and the machine learning potential is the deep potential range corrected (DPRc) model[33-34] implemented in DeePMD-kit.[35] The DPRc model is an extension of the DeepPot-SE model[36] to correct both the QM/QM and nearby QM/MM interactions in a manner that conserves energy as MM atoms approach (or leave) the vicinity of the QM region. The mathematical form of the DPRc correction can be found in refs. 33 and 34. The fitting network in the present work is composed of 3 hidden layers using 240 neurons/layer, and the output is a scalar correction to the energy. The filtering network is composed of 2 hidden layers using 25 and 50 neurons, and the output is 100 neurons of the "embedding matrix". The "coordinate matrix" (the input layer of the filtering network) scales the inverse distances between the QM and MM atoms, such that neural network corrections smoothly approach zero at a distance of 6 Å. Each layer was activated with the hyperbolic tangent function. We parametrized the neural network parameters to simultaneously reproduce the PBE0/6-31G* QM/MM energies and forces of all 4 systems (unmodified MTR1 and the 3 variants). Separate trainings were performed with O^6mG and ab^{6}G using the same training procedure. The parametrizations were performed using the DP-GEN software.[37] The neural network parameters were updated from 100,000 steps of Adam stochastic optimization with an initial learning rate of 0.001. A detailed description of the loss function can be found in ref. 35. In the present work, the loss function contains contributions from energies and forces. The prefactor associated with each property has a dependence on the learning rate. The initial and final prefactors on the energy are 0.02 eV^{-2} and 1.0 eV^{-2}, respectively. The initial and final prefactors on the forces are 1000 $eV^{-2}Å^2$ and 1.0 $eV^{-2}Å^2$. Parametrizations were repeated 4 times with different random number seeds to produce 4 sets of trial neural network parameters. The initial parameterization was performed on 3,200 structures extracted from the DFTB3/3ob QM/MM umbrella sampling. This comprised 8 training iterations. The parameters were refined through 10 iterations of active learning based on convergence of the training error and model deviation as shown in Figures S6 and S7. The active learning cycles restart the umbrella sampling of 896 images for 1 ps using 1 of the neural network parameter sets. These images correspond to 32 windows from 7 iterations of the string method for each of the 4 systems. The force differences in the 4 neural network parameter sets are compared from the new sampling. If the standard deviation in an atomic force between the models was between 0.08 and 0.25 eV $Å^{-1}$, then the structure was designated a "candidate". Up to 1,000 candidate structures were selected at random to be labelled; i.e., *ab initio* PBE0/6-31G* single point evaluations were performed. The newly labelled data was appended to the available reference data used to update the parameters in the next active learning iteration. After training the ΔMLP_{PBE0} correction, the string method was reperformed with 1 of the neural network parameter sets, selected at random.

Alchemical free energy simulations

We calculate apparent pK_a shifts of the unmodified MTR1, C10(n1c,c5n), C10(n1c), and A63(n7c) variants. The pK_a shifts, ΔpK_a, are relative to the pK_a of the nucleotide in the aqueous phase.

$$\Delta pK_{a,A} = \frac{\Delta G_{MTR1}(AH^{+} \rightarrow A) - \Delta G_{aq}(AH^{+} \rightarrow A)}{RT\ln(10)} \tag{S 1}$$

Additionally, we calculate the apparent pK_a shift of the A63(n7c) variant at the N1 position of nucleotide 63. The analogous calculation for the unmodified ribozyme was performed in previous work.[20]

The free energy differences are calculated from alchemical free energy (AFE) simulations. In these simulations, the atomic charges of the nucleotide of interest are modified such that the λ = 0 and λ = 1 states correspond to protonated and deprotonated nucleotides, respectively. The deprotonated nucleotide partial charges were determined from RESP charge fitting procedure,[25] where the potential was calculated at HF/6-31G*. The alchemical transformation was modelled with the modified SSC(2) softcore potential,[18] where the nucleotide was included in the common-core region and the proton was included in the softcore region. The implementation of the SSC(2) softcore potential in the pmemd program includes options which control the λ-dependence of the bonded terms crossing the common-core and softcore regions. Specifically, we set the option gti_add_sc=25 which scales the energy of those torsions describing the rotation around a single-bond.[38] This scheme has been found to enhance the sampling and improve the convergence of the calculated free energy.[38] The transformation involved 25 states that uniformly discretize the alchemical (λ) dimension. The systems were prepared in accord with our ProFESSA free energy workflow.[39] Production sampling was obtained in the *NPT* ensemble at 298 K and 1 atm using the alchemical enhanced sampling (ACES) method.[38] Each state was sampled for 5 ns and Hamiltonian replica exchange attempts were made every 20 fs. The production sampling was repeated 3 times with different thermostat random number seeds. Free energies were calculated from the MBAR method[28] implemented in FE-ToolKit.[31, 40]

Constructing activity-pH profiles

The theoretical activity-pH profile of each system was computed based on the DFTB3/3ob+ΔMLP$_{PBE0}$ free energy barrier and the predicted pK_a of the general acid (A) using a single pK_a model for the observed rate:

$$k_{obs} = k_{int}/(1 + 10^{pH - pK_{a,A}}) \tag{S 2}$$

where the intrinsic rate (k_{int}) is estimated from transition state theory as

$$k_{int} = \kappa \frac{k_B T}{h} e^{-\frac{\Delta G^{\ddagger}}{k_B T}} \tag{S 3}$$

and the pK_a of the general acid is estimated as

$$pK_a = pK_a^{expt.} + \Delta pK_{a,A}. \tag{S 4}$$

R is the gas constant, T=298 K, K_B is the Boltzmann constant, h is the Planck constant, and κ is a transmission coefficient. In this work κ is assumed to be 1.

3D-RISM calculation

A 3D-RISM calculation was performed to explore potential Mg^{2+} ions binding sites near the C10(n1c) system active site that could plausibly promote an alternative mechanism of proton transfer. The calculation was performed was outlined in previous work.[41-42] First, DRISM was performed with the rism1d program with 32,769 grid points and 0.025 Å spacing to determine the site-site solvent susceptibilities. The solvent was maintained at constant density of 55.296 M SPC/E water with 10 mM Mg^{2+}, 140 mM Na^{+}, and 160 mM Cl^{-}. The modified direct inversion of iterative subspace (MDIIS) method was used to iteratively solve the DRISM equation with PSE2 closure to a residual tolerance of 10^{-12} at 298 K and a dielectric constant of 78.497 for bulk water. The 3D-RISM calculation was performed with the 3D-RISM interface to sander under periodic boundary conditions using PSE1 closure with 0.5 Å grid spacing within the box dimensions used for MD simulations.

Data Availability

Modified nucleotide parameters and coordinates will be made available on the York Group Gitlab page: https://gitlab.com/groups/RutgersLBSR

SUPPLEMENTARY RESULTS

The active site hydrogen bonding pattern is maintained upon atomic mutagenesis in MD simulations

The atomic mutants used in this study were carefully selected to introduce pK_a perturbations without interrupting the Watson-Crick face of the C10 and A63 nucleotides, as these form crucial hydrogen bonds to hold the ligand in the active site. To ensure the integrity of these interactions upon modification, we performed 50 ns of MD simulations with the C10(n1c,c5n), C10(n1c), and A63(n7c) containing systems and measured the hydrogen bond lengths as well as nucleophile to electrophile distance formed by these nucleotides and O^6mG. These are compared to the first 50 ns of production simulation that was performed for the unmodified ribozyme as shown in Figure S3. We find that the average values of these distances are comparable before and after each modification, suggesting that the modifications do not interrupt the integrity of the active site.

Molecular dynamics simulations of MTR1 with the ab^{6}G cofactor

Molecular dynamics simulations were conducted to study the accommodation of ab^{6}G into the MTR1 active site and prepare the system for QM/MM simulations. As shown in Figure S4A, the root mean square deviation of the position of the heavy atoms with respect to the starting structure is slightly higher in some cases than in simulations of the O^6mG containing MTR1 (< 5 Å). This is to be expected, as the system was crystalized in the presence of O^6mG, and ab^{6}G introduces more rotatable bonds. In particular, it was important to establish that these rotations do not hamper the inline attack angle (denoted O6-C10-N1), which is well maintained as shown in Figure S4B. We measured the dihedral angles describing rotations of the 4-aminomethyl-benzyl group with respect to the plane of the ab^{6}G (C1-O6-C10-C11), and rotation of the amine with respect to the benzene ring (C11-C14-C17-N18). The atom numbers are given in Figure S4C. In general, the sidechain is angled down below the plane of the active site, and the amine samples a range of dihedrals, interacting transiently with A9, G8, and solvent. Despite these rotations, the average inline attack angle was 164° across the 5 simulations, indicating good alignment of the nucleophile, electrophile, and leaving group.

Structural differences between C10 and C10(n1c,c5n)

The pK_a of C(n1c,c5n) in solution is 0.5 units lower than C, but the pK_a in MTR1 both from experimental fits and alchemical free energy simulations suggest this pK_a to be shifted slightly above the unmodified ribozyme. The C10(n1c,c5n) substitution maintains the Watson-Crick face, but it introduces an additional hydrogen bond donor at the N5 position. 50 ns of MD simulation was performed for the C10(n1c,c5n) substituted system to investigate this effect on the active site structure compared to the unmodified ribozyme. Based on these simulations the difference in pK_a shifting could plausibly arise from a variation in the hydrogen bonding pattern between C10(n1c,c5n) and the phosphate of A9, as shown in representative structures in Figure S5. This hydrogen bonding is dynamic, with either N5 (shown in Figure S5B) or the exocyclic amine (N4, equivalent to the interaction shown in Figure S5A) able to hydrogen bond to A9:*pro*R. When the exocyclic amine of C10(n1c,c5n) is hydrogen bonding to A9:*pro*R, the hydrogen on N5 can form an additional hydrogen bond with the A9:O5′. Thus C10(n1c,c5n) forms a stronger interaction than the unmodified ribozyme.

Convergence of the machine learning potential training error

A machine learning correction to the DFTB3/3ob potential was trained to replicate PBE0/6-31G* energies and forces for the MTR1 reaction. As demonstrated in Figure S6A, the final model greatly improves the energy and force predictions for the training set. We then validated the performance of the final model on a test set of 500 structures generated from additional simulations along the reaction path that had not yet been trained on. As shown in Figure S6B, the performance of the model for the test set is comparable to the training set, thus justifying termination of the training procedure.

Previous wok by our group has shown 10 iteration of active learning to be sufficient to reach convergence within the desired accuracy without overtraining.[33] We find 10 rounds of active learning training to be sufficient for this application as >99% of the training data consistently produced model deviation of the forces less than or equal to 0.08 eV/Å, which was the cutoff value in selecting structures as candidates for PBE0/6-31G* level energy and force evaluations. This is demonstrated in Figure S7 along with the convergence of the error between machine learning predicted and target forces and energies for the training set.

Confirmation of step-wise mechanisms

Path-finding simulations using the surface accelerated string method at both the DFTB3/3ob and DFTB3/3ob+ΔMLP$_{PBE0}$ level converged upon a stepwise mechanism where proton transfer precedes methyl transfer for the unmodified ribozyme, C10(n1c,c5n), and A63(n7c) with both cofactors. Simulations of the C10(n1c) system converged upon the opposite mechanism. To ensure that the opposite mechanism for each system was indeed unfavorable, DFTB3/3ob+ΔMLP$_{PBE0}$ QM/MM simulations with *O*^{6}mG bound were performed on a regular grid for 25 ps, the results of which are shown in Figure S8. In each case, the mechanism deduced from the pathfinding is confirmed to be the minimum free energy path. Therefore, further production simulations and analysis were only performed on that path as the DFTB3/3ob+ΔMLP$_{PBE0}$ path is meant to very closely resemble PBE0.

Structural impacts of charge distribution with A63(n7c) substitution

The enhanced nucleophilicity of A63(n7c):N1 not only upshifts its pK_a, but it could plausibly influence the distribution of charge across the nitrogen atoms in the molecule. This would be especially relevant in the product state when nucleotide 63 is positively charged. We investigated the effect this could have on the length of the hydrogen bond between the A63(n7c) exocyclic amine (H61) and the ligand N7 by measuring the hydrogen bond distance and calculating the Mulliken charge on the participating heavy atoms across the 4 production trial trajectories. We observed that the hydrogen bond is generally shorter than in the unmodified ribozyme over the course of the reaction as shown in Figure S9A. This appears to arise from A63(n7c):N6 experiencing more positive charge than the unmodified A63 as shown in Figure S9B, presumably because N1 becomes more electronegative. This allows the A63(n7c) exocyclic amine to be a better hydrogen bond donor and strengthen this structurally important interaction.

Kinetic and structural impacts of the alkyl donor

The computational work done previously was conducted with *O*^{6}mG as an alkyl donor; however the experimental rate of ab^{6}G was found to be faster and was used to perform the experiments in this work. We sought to compare the free energy barrier of *O*^{6}mG and ab^{6}G at the DFTB3/3ob+ΔMLP$_{PBE0}$ level and confirm that the mechanisms are consistent. A separate ΔMLP correction was trained and

200 ps DFTB3/3ob+ΔMLP$_{PBE0}$ production simulations were performed. The resulting free energy barrier is compared with O^6mG for the unmodified ribozyme in Figure S10A and a representative structure is shown in Figure S10B. The free energy profiles for the unmodified ribozyme and A63(n7c) were similar to the profiles for methyl transfer, but with a lower TS2 barrier (Figure S11). The C10(n1c) barrier was only slightly reduced because, without proton transfer, the only benefit of the electron withdrawing 4-aminomethyl-benzyl group is making the carbon electrophile stronger.

Interestingly, QM/MM simulations of the C10(n1c,c5n) containing MTR1 with O^6mG as the alkyl donor produced a rate determining barrier higher than that of the unmodified ribozyme, whereas the trend is reversed with ab^{6}G (Figure S11). Over the course of the reaction, the hydrogen bond between the C10 exocyclic amine and O6 leaving group becomes shorter as negative charge collects on O6, and we hypothesize that with O^6mG as the alkyl donor this stabilizing interaction is weaker for C10(n1c,c5n) due to its additional interactions with the A9 phosphate (Figure S5). In the presence of ab^{6}G, the dynamic nature of the rotatable amine generally lengthens the hydrogen bond between the C10 exocyclic amine and the A9 phosphate, thus plausibly reducing the energy required to bring the exocyclic amine closer to O6 in TS2.

Alternative proton transfer mechanisms for C10(n1c)

According to DFTB3/3ob+ΔMLP$_{PBE0}$ QM/MM simulations of the C10(n1c) system, the rate would be undetectable; however, a pH independent rate of ~10^{-4} / min was observed experimentally. We investigated 3 alternative mechanisms compared to the baseline proton transfer from C10(n1c):N3 at the DFTB3/3ob level. These included U45:N3, C10(n1c):N4, or A63:N6 as the proton donor to hypothesize the source the discrepancy in the predicted rate (Figure S12A-D). The simulations were first performed using the O^6mG cofactor, and the resulting free energy profiles are shown in Figure S12E. Previous experimental work has demonstrated that the C10U mutation completely inactivates the ribozyme,[19] which was consistent with QM/MM simulations.[20] The only hydrogen bond lost upon C10U mutation is between C10:N4 and O^6mG, thus we initially hypothesized this to be the proton donor for C10(n1c) since it would not be available for C10U. However, the free energy profiles reject this notion and suggest U45:N3 to be the best general acid.

Based on the simulations of MTR1 with ab^{6}G bound and the crystal structure by Höbartner and coworkers, we wondered whether interactions of the positively charged NH_3 group with A9, and subsequent distancing of N4 from A9:*pro*R could make the C10(n1c):N3 and N4 proton transfers more favorable. In the simulations of the unmodified MTR1 in which C10 is positively charged, the NH_3 group is dynamic and transiently interacts with A9, and the A9:*pro*R interaction is productive in upshifting the C10 pK_a. However, for proton transfer to occur from C10(n1c), the nucleobase becomes negatively charged and would benefit from a downshift in pK_a. Therefore, we repeated the DFTB3/3ob simulations with ab^{6}G bound, and the resulting free energy profiles are shown in Figure S12F. While each of the barriers where suppressed, U45:N3 proton transfer remained the most favorable. Given the high barrier of the previous C10U calculations, we remained hesitant to rule out proton transfers from C10(n1c), and next investigated potential rate enhancements from metal ion binding.

In MD simulations there are consistently two territorial Na^+ ions above the phosphate of A9 and between U45:O4 and the phosphate of G41. Na^+ was seldom observed near C10:O2; however a Mg^{2+} ion was observed there in the crystal structure of Höbartner and coworkers with ab^{6}G bound.[21]

Furthermore, the U45:O4 site is occupied by a barium ion in the structure of Deng et al.,[19] and by a thallium(I) ion in the structure by Höbartner and coworkers (PDB code 7Q82).[21] We hypothesized that metal ion binding at these sites would enhance the C10(n1c):N3, C10(n1c):N4, and U45:N3 proton transfer mechanisms. We performed a 3D-RISM calculation on the C10(n1c) structure to assess likelihood of metal ion binding at these sites. As shown in Figure S13A, the most probable Mg^{2+} binding sites near the active site are around the A9 phosphate and U45:O4, while Mg^{2+} binding at C10:O2 was much less probable. Based on these observations, we performed simulations with Na^+ bound at C10:O2, Mg^{2+} at A9:*pro*R, and Mg^{2+} at U45:O4. Since these ions do not interact with A63, the A63:N6 proton transfer mechanism was not considered further, and U45:N3 proton transfer was only considered with Mg^{2+} at U45:O4 since the other two sites would not directly impact this mechanism. The resulting free energy profiles are given in Figure S13B-D.

Despite further lowering the alkyl transfer barrier, metal ion binding did not make the proton transfers from C10(n1c) sufficiently favorable. Meanwhile, Mg^{2+} binding at U45:O4 made this already promising mechanism more favorable. Based on this data, we refined the U45:N3 proton transfer free energy profiles at the PBE0 level. Upon switching to the high level Hamiltonian, proton transfer from U45:N3 with only Na^+ bound at O4 yielded a barrier of 32.6 kcal mol^{-1}. With Mg^{2+} bound at O4, this was reduced to 25.9 kcal mol^{-1} (Figure S14A-B), which translates to approximately $3.7x10^{-5}$ min^{-1} in intrinsic rate based on transition state theory, close to the observed rate (Table 1). A mechanism based on proton transfer from U45:N3 would be likely to be pH independent over the range investigated of pH 6-8. Given the short simulation time and varied conditions, it is difficult to compare this barrier to the other MTR1 substitutions, however this suggests a plausible alternative mechanism for C10(n1c) that could yield an experimentally detectable rate.

This mechanism would likely also be available for C10U, assuming this mutation allows for proper folding and cofactor binding. We evaluated the Mg^{2+} assisted U45:N3 proton transfer mechanism for C10U at the PBE0 level, and the barrier was increased to 28.0 kcal mol^{-1}, which equates to an approximately 10 fold decrease in the predicted intrinsic rate due to the clash between C10U:O4 and the leaving group, O6. Results of the PBE0 simulations are shown in Figure S14A. It is possible that C10U could be theoretically reactive through this pathway, however this still may fall outside of the experimental detection limit, especially if the C10U modification weakens substrate binding. Although it is difficult to confirm whether this is the definitive pathway of C10(n1c), it is clear that regardless of any proton transfer, the barrier for C10U is expected to be higher than C10(n1c) by ~2-3 kcal mol^{-1}. This is demonstrated by the 1-dimensional free energy profile corresponding to strictly alkyl transfer without any additional metal ions in Figure S14C.

Evaluation of a two pK_a model

In addition to the current work, activity-pH profiles by Deng and coworkers[19] and Scheitl and coworkers[21] appeared to show a downturn at low pH, thus we hypothesized that perhaps a pK_a of ~5 could arise from protonation of the A63:N1 nucleophile blocking the reaction. However, alchemical free energy simulations in previous work suggested a downshifted pK_a of 2.8, which would not be consistent with this hypothesis.[20] Here we compute the analogous pK_a of A63(n7c) at the N1 position to be 4.8 in the ribozyme, downshifted from the solution value of 5.3. When these values are used in a two pK_a model, the curve is almost indiscernible from a single pK_a model in the experimentally relevant range of 5.5 to 8.5. Therefore, it is plausible that at low pH the nucleophile may be blocked, but this is not mechanistically important under the experimental

conditions considered. This is demonstrated in Figure S16, where the observed rate is normalized using Equation S5 such that maximum value of the MTR1 single pK_a fit (black solid line) is aligned to the maximum value of the experimental MTR1 fit (grey solid line).

$$K_{norm,MTR1} = K_{obs,sim}\left(\frac{\max\,(K_{obs,exp})}{\max\,(K_{obs,sim})}\right) = K_{obs,sim} \cdot 0.0017$$

S 5

SUPPLEMENTARY FIGURES

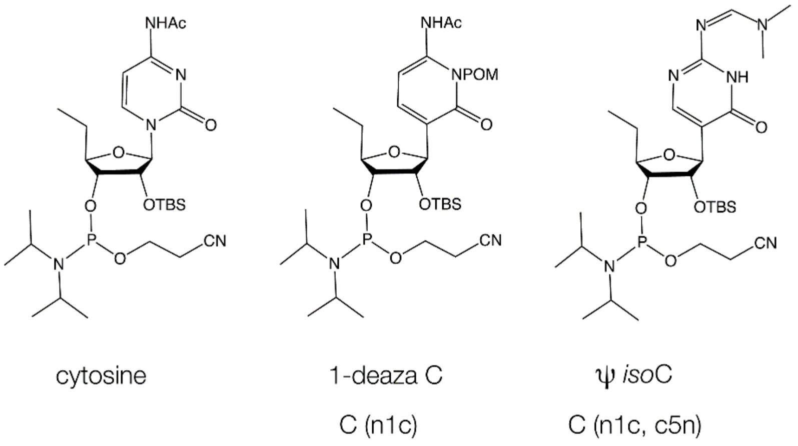

Figure S1. Phosphoramidites used to synthesise atomic variants of MTR1 at C10.

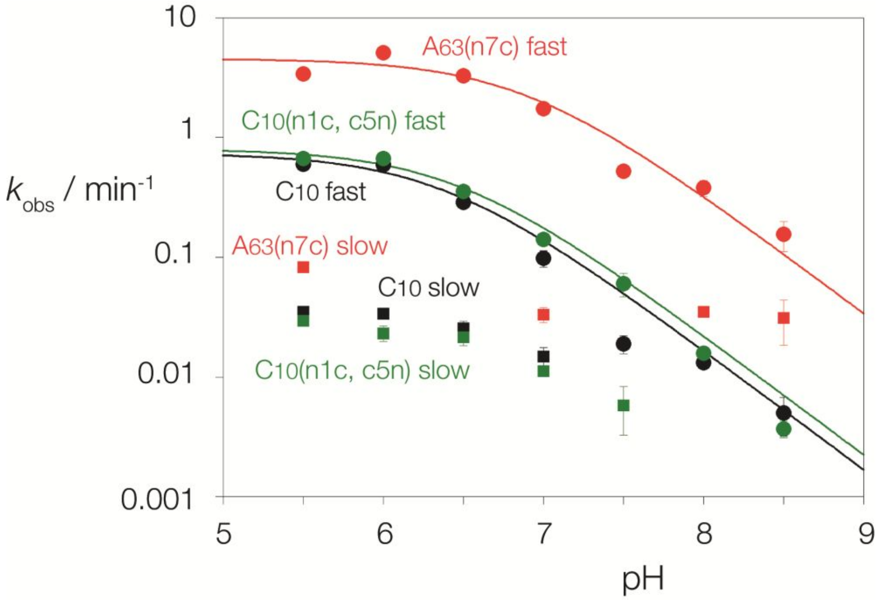

Figure S2. Fast and slow rate constants for MTR1 and variants as a function of pH. Experimental reaction progress was fitted to two exponential functions; here we have separated the data into the two rates for each. Circles are the fast rates and squares are the slow rates. Where there is no square plotted (such as for C10 at pH 8.5) the reaction progress was well fitted by a single exponential. Black and green data are MTR1 with C10 and C10 (n1c, c5n) substitution, and red data are MTR1 A63(n7c).

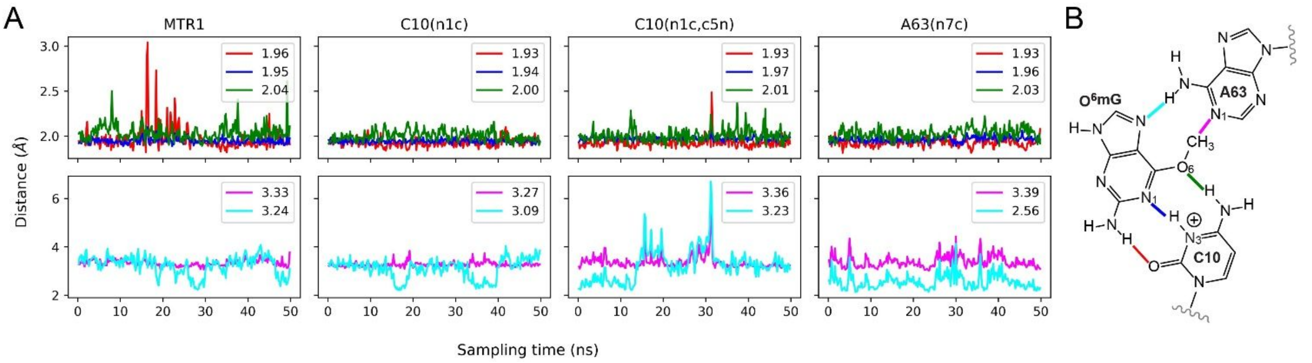

Figure S3. Analysis of key interactions between C10 and A63 and *O*[6]mG from 50 ns of MD simulations. **(A)** 5000 trajectory frames were output, and a 20 frame running average tracking 5 indicated distances is plotted for clarity. The mean from each simulation is given in the legend. The color of each line corresponds to the highlighted interactions in **(B)**. Note that the exocyclic amine of *O*[6]mG experiences occasional flips, thus the red line corresponds to the hydrogen bond with the closer of the two hydrogens for a given frame.

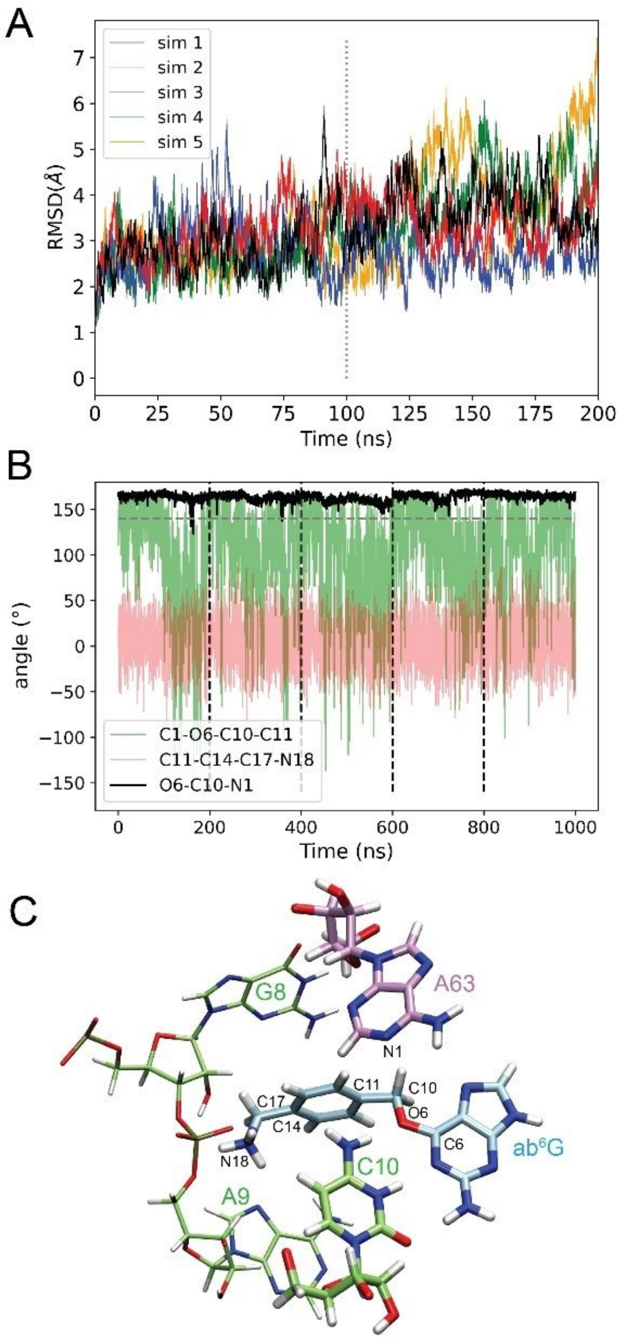

Figure S4. Analysis of MD simulations of MTR1 with the ab^{6}G cofactor. **(A)** Root mean square deviation (RMSD) analysis of heavy atoms aligned to the first frame of 5 independent molecular dynamics simulations of MTR1 with ab^{6}G bound. Simulations 2 through 5 were initiated from structures at t = 25, 50, 75, and 100 ns from simulation 1 (black). **(B)** Impact of key ab^{6}G sidechain dihedral angles (red and green) on the nucleophilic inline attack angle (black). The grey dashed line indicates that angles above 140° are deemed "catalytically fit", black dashed lines indicate the beginning of independent simulations, and angles plotted are a 0.2 ns running average for clarity. **(C)** Atoms involved in the angles plotted in (B) (black labels). The depicted C6-O6-C10-C11, C11-C14-C17-N18, and O6-C10-N1 angles are 165°, -18° and 171° respectively.

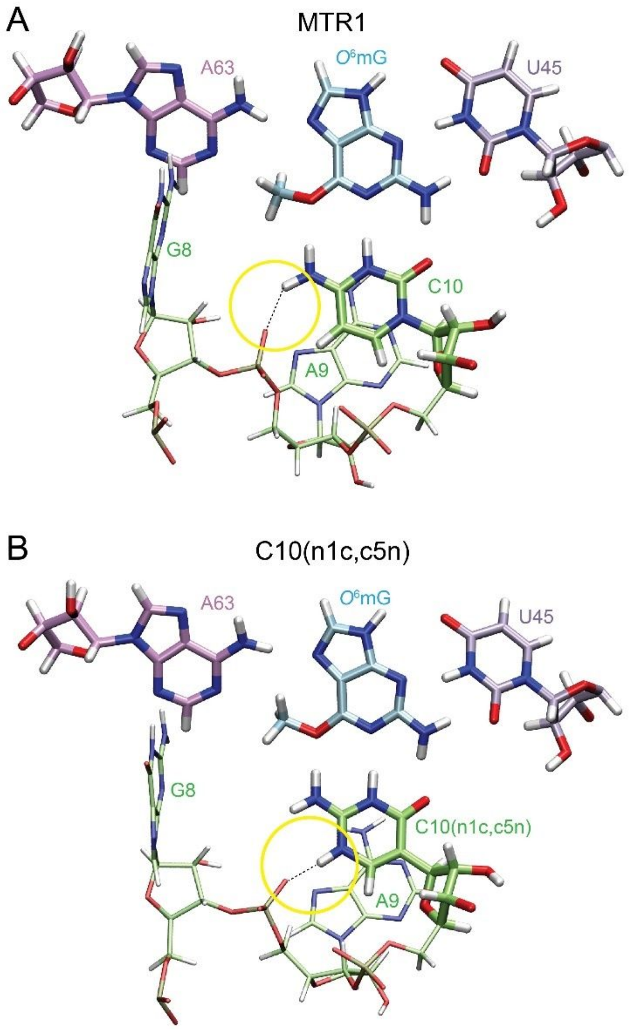

Figure S5. Representative structures depicting interactions made by nucleotide 10 with phosphate of A9 in **(A)** MTR1 and **(B)** C10(n1c,c5n).

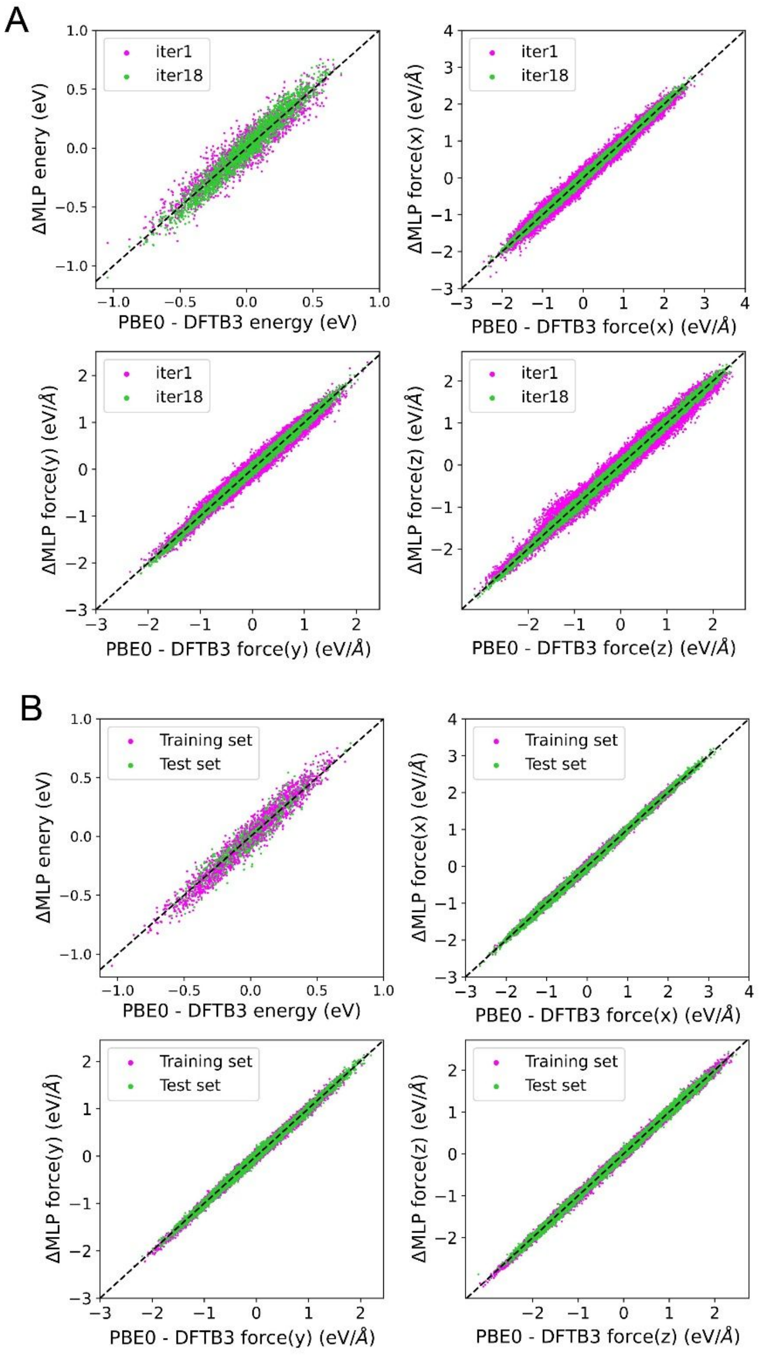

Figure S6. Analysis of errors in the training of a DFTB3/3ob+ΔMLP_{PBE0} model in which ΔMLP (y-axis) is trained to replicate the difference between PBE0/6-31G* and DFTB3/3ob (x-axis) energies and forces. **(A)** The error for the training set is analysed after the first (iter1, pink) and final (iter18, green) iteration of training. **(B)** The error for the training set (pink) after the final iteration of training is compared to that of a test set containing 500 structures (green). The energies are taken as a difference from the mean to aid comparison as they differ within a constant.

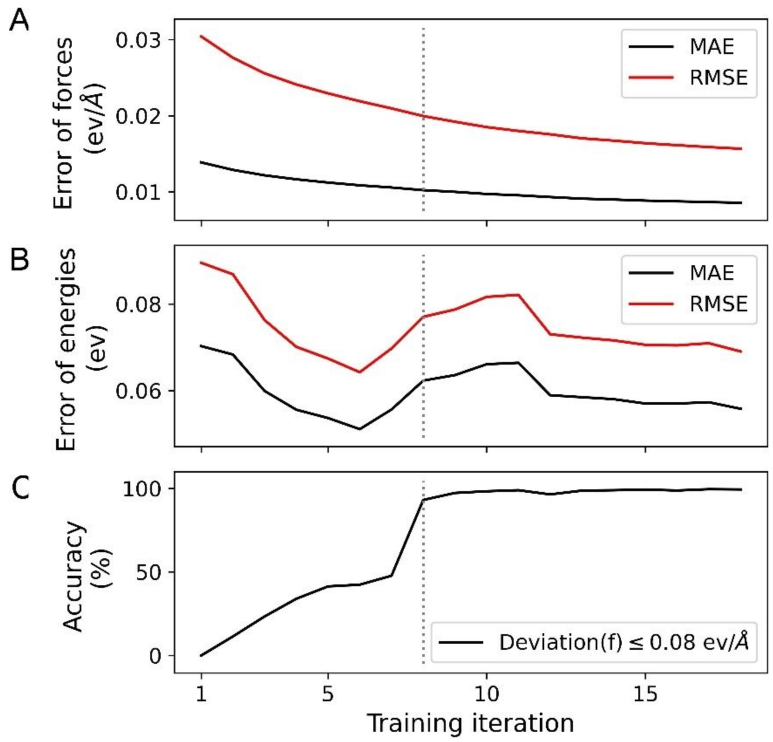

Figure S7. Convergence of the root mean square error (RMSE) and mean average error (MAE) between the machine learning correction to the forces **(A)** and energies **(B)** compared to the computed difference at the DFTB3/3ob and PBE0/6-31G* level. **(C)** The percentage of training structures deemed accurate at each iteration with deviation in forces between the 4 trained models falling at or below 0.08 eV/Å. A vertical dotted line indicates the beginning of the active learning procedure. The energies are taken as a difference from the mean to aid comparison as they differ within a constant.

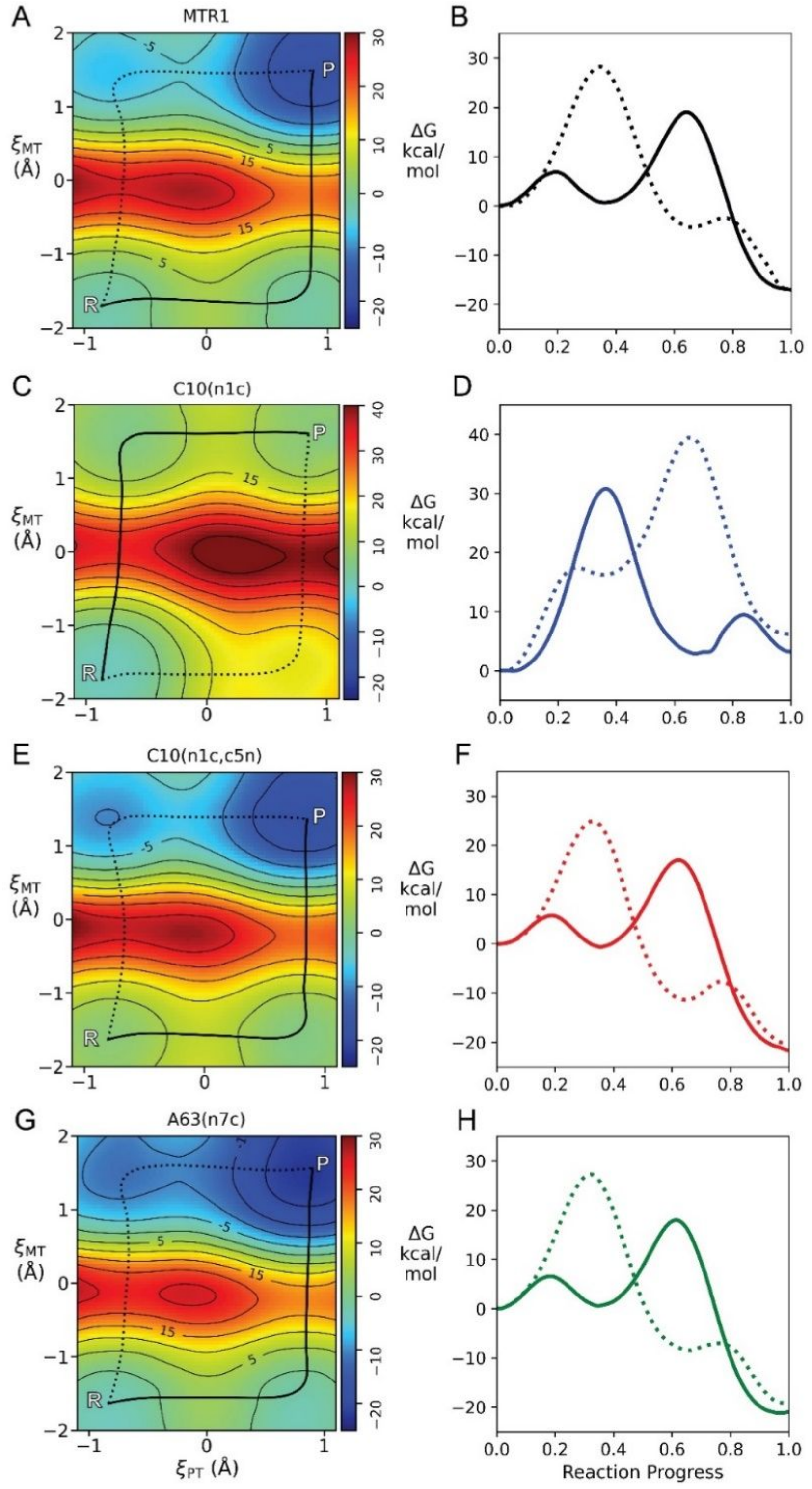

Figure S8. 2D free energy landscapes and corresponding reaction free energy profiles of the studied variants; **(A,B)** MTR1, **(C,D)** C10(n1c) **(E,F)** C10(n1c,c5n) and **(G,H)** A63(n7c). Surfaces are constructed from 25 ps of sampling at the DFTB3/3ob+ΔMLP$_{PBE0}$ level with *O*^{6}mG as the alkyl donor. Two alternate paths are illustrated on each surface where the minimum free energy path is denoted with a solid line, and the alternative with a dotted line. Simulations were performed for 25 ps on a regularly spaced 0.2 Å grid.

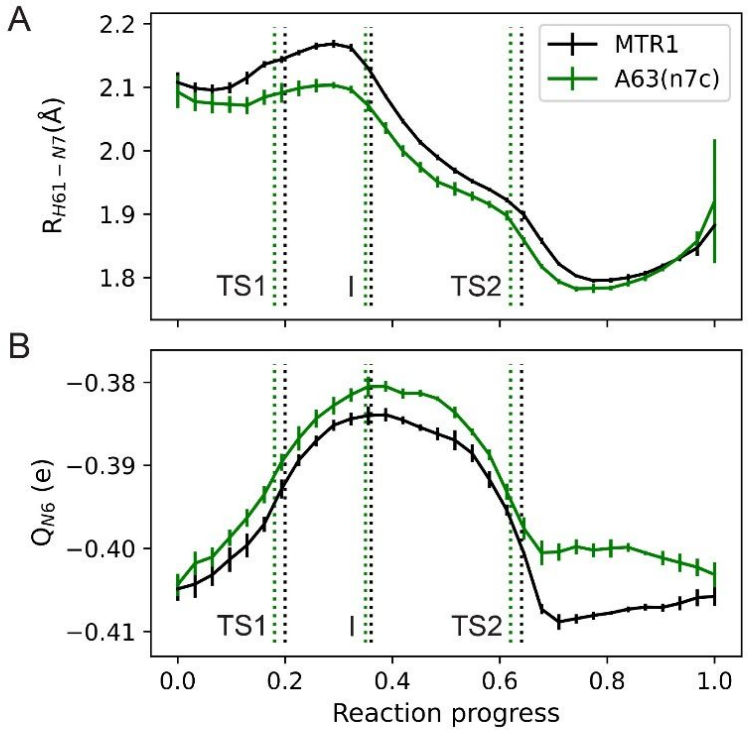

Figure S9. Correlation of hydrogen bond distance between the nucleotide 63 exocyclic amine (H61) and *O*[6]mG:N7 **(A)** with Mulliken charges of A63:N6 **(B)** over the course of the reaction for the MTR1 (black) and A63(n7c) (green). Values are the averages within each umbrella window. Error bars represent the standard deviation over 4 independent 200 ps production runs. Vertical dotted lines indicate the location of stationary points as deduced from the free energy profile.

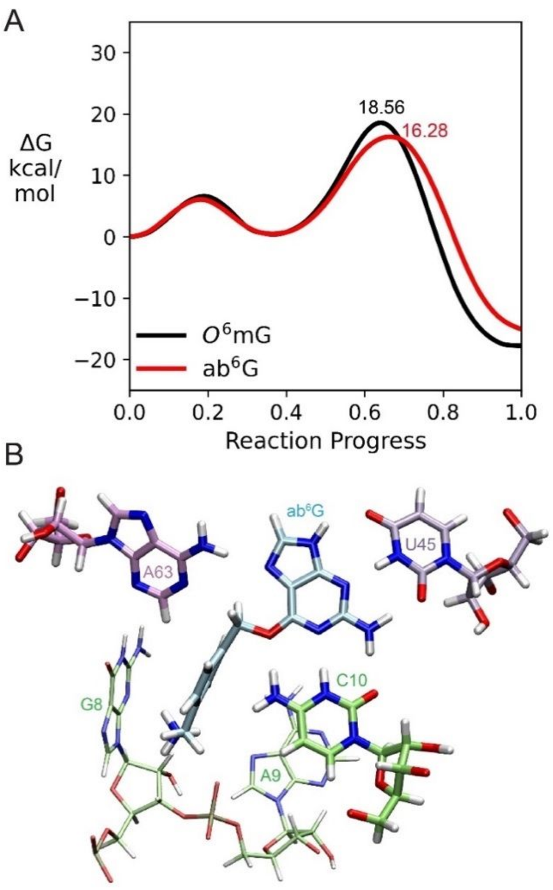

Figure S10. Effect of alkyl donor group on the MTR1 reaction barrier and the structure of the active site. **(A)** Free energy profile of the MTR1 reaction at the DFTB3/3ob+ΔMLP$_{PBE0}$ level with O^6mG (black) and ab^{6}G (red) as the alky donor group with associated free energy barrier. **(B)** Representative structure of MTR1 with ab^{6}G bound.

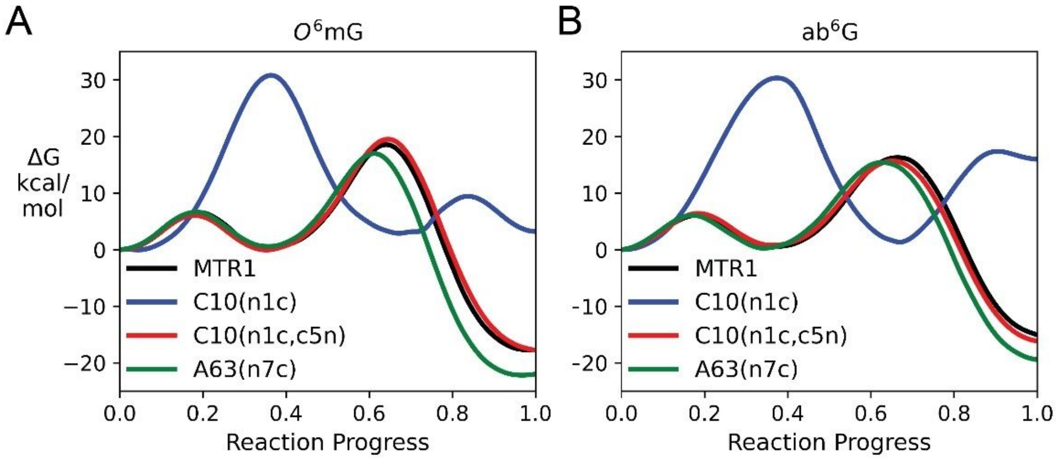

Figure S11. Comparison of the free energy profiles of all studied MTR1 variants with **(A)** O^6mG and **(B)** ab^{6}G as the alkyl donor at the DFTB3/3ob+ΔMLP$_{PBE0}$ level.

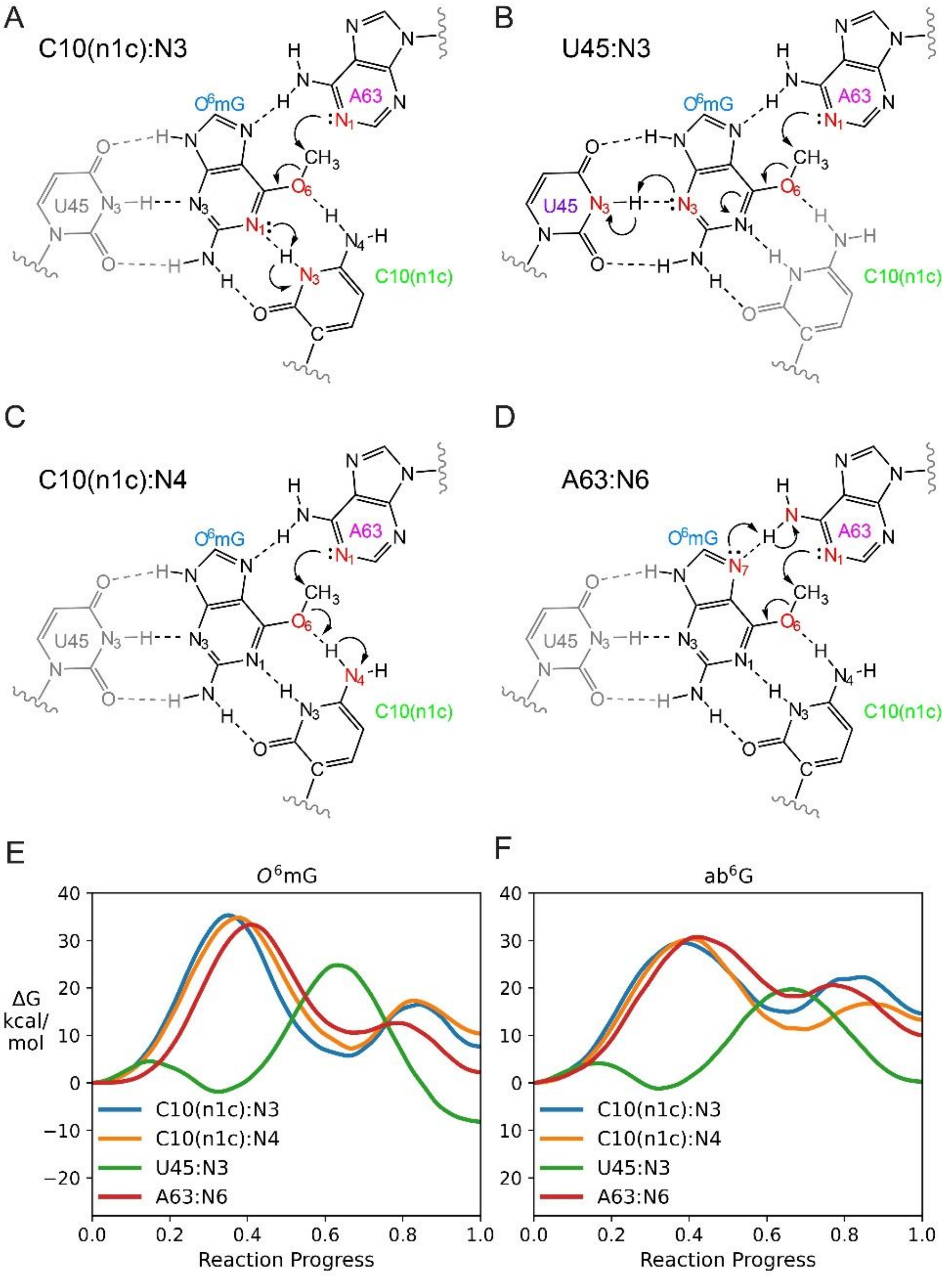

Figure S12. Investigation of alternate proton transfer mechanisms for the C10(n1c) reaction. (**A-D**) Proposed reaction mechanisms for the proton transfer step where source of the proton are: **(A)** C10(n1c):N3, **(B)** U45:N3, **(C)** C10(n1c):N4, and **(D)** A63:N6. (**E-F**) Free energy profiles for each mechanism obtained with **(E)** O^6mG and **(F)** ab^{6}G bound, obtained using DFTB3/3ob level of theory. In each profile, the high barrier step corresponds to methyl transfer, and the low barrier step to proton transfer.

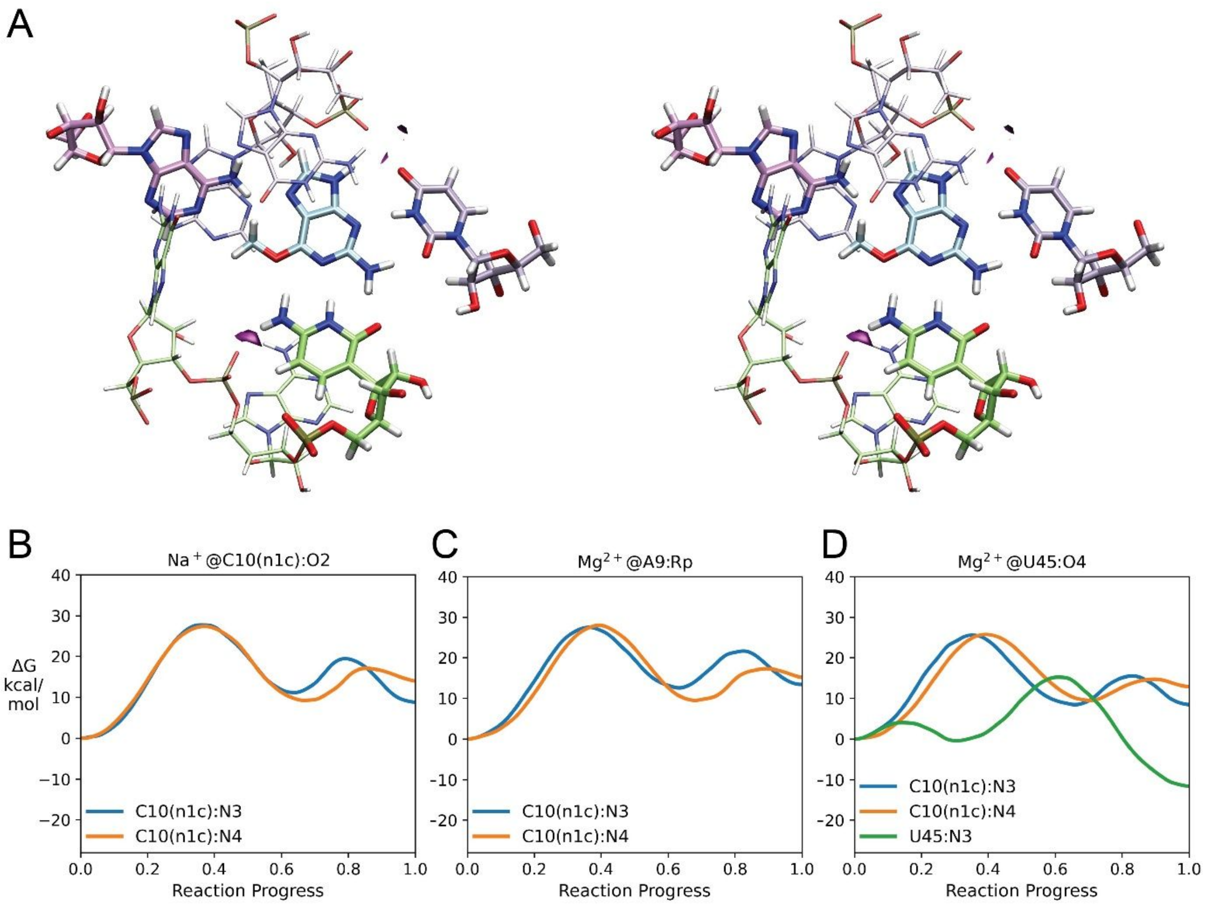

Figure S13. Impact of metal ion binding on proposed proton transfer mechanisms. **(A)** Mg^{2+} ion density (magenta) predicted from 3D-RISM calculations depicting the 72% contour level around the C10(n1c) active site (stereo image). Most probable Mg^{2+} binding sites are around A9:Rp and U45:O4. **(B-D)** Free energy profiles of the explored proton transfer mechanisms with introduction of metal ions (Na^{+} or Mg^{2+}) directly coordinating specific sites. Simulations were performed with ab^{6}G bound at the DFTB3/3ob level of theory. **(B)** Na^{+} at C10(n1c):O2, **(C)** Mg^{2+} at A9:Rp, and **(D)** Mg^{2+} at U45:O4. In each profile, the high barrier step corresponds to methyl transfer, and the low barrier step to proton transfer.

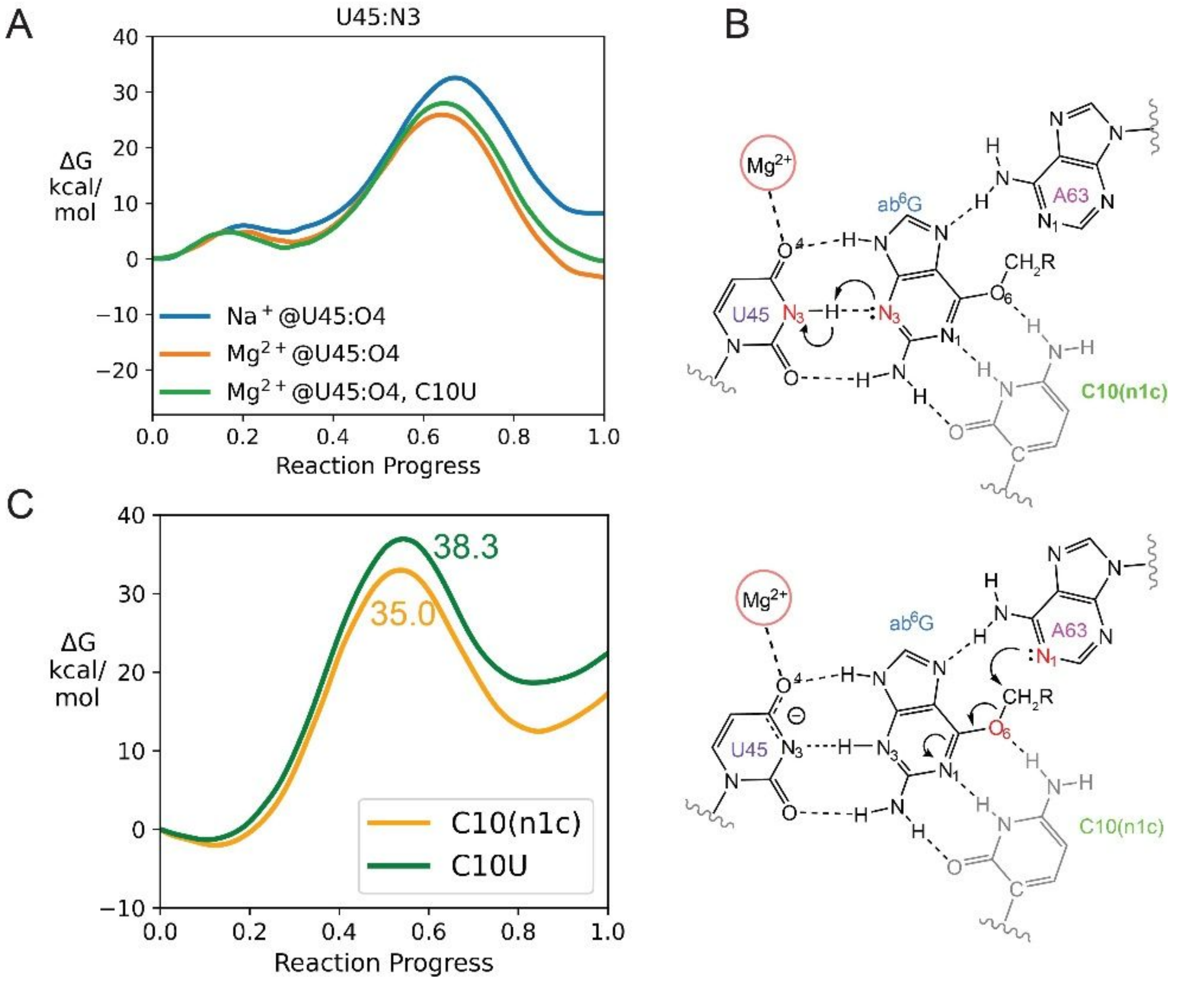

Figure S14. Plausible alternative mechanism of alkyl transfer for C10(n1c) with proton transfer from U45:N3 investigated with high level theory. **(A)** Free energy profiles when Na^+ and Mg^{2+} are coordinated to U45:O4 obtained at the PBE0 level of theory. Na^+ is consistently observed at this site and is implicit in all proposed mechanisms. **(B)** Deduced U45 general acid catalyzed mechanism reliant on Mg^{2+} binding plausibly explaining the detectable activity of C10(n1c). **(C)** 1-dimensional free energy profiles representing alkyl transfer for C10(n1c) and C10U without proton transfer obtained at the PBE0 level of theory. Free energy barriers are given in kcal mol^{-1}.

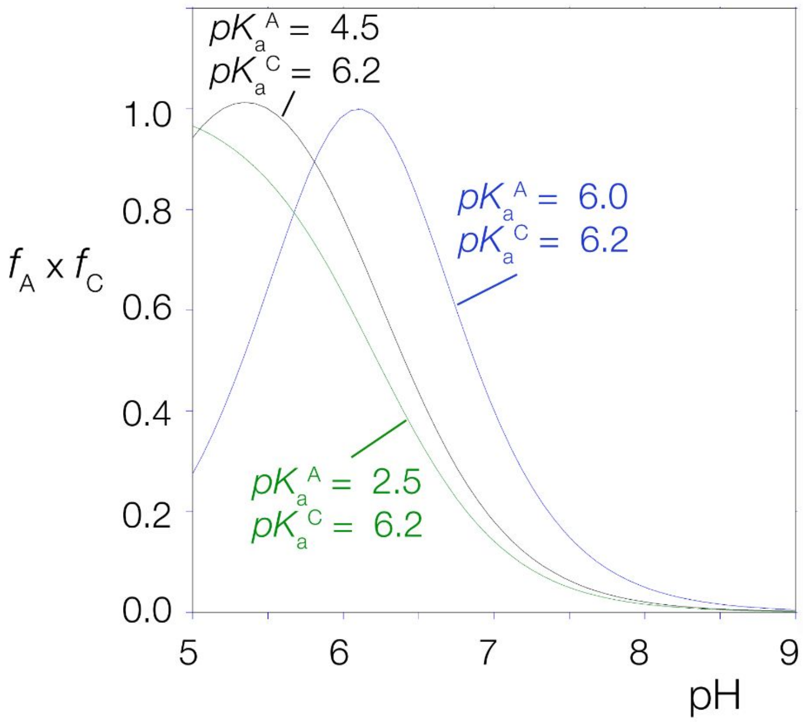

Figure S15. Calculated pH dependence of the rate of alkyl transfer by MTR1 as a function of the pK_a of the general acid C10 and the nucleophile A63. The calculations are based upon two ionizations. The active ribozyme requires C10 to be protonated (fraction f_C), and A63 to be unprotonated (fraction f_A), and the fraction of active ribozyme at any pH will be proportional to f_A x f_C . This is plotted as a function of pH, and will be proportional to the variation in reaction rate with pH. The pK_a of C10 was fixed at 6.2, and three values of the pK_a of A63 were used to calculate the pH dependence. A pK_a = 4.5 (black) is close to that of free adenine, and leads to a profile similar to that of the unmodified ribozyme. A pK_a = 6.0 (blue) corresponds to a rise of 1.5 units, as expected for A (n7c). Lastly, a pK_a = 2.5 (green) leads to an maximum of f_A x f_C that is off the pH scale ; computation indicates that in the environment of the ribozyme the pK_a of A63 is actually substantially lowered. Raising the pK_a = 6.0 (blue) as expected for A (n7c) leads to a readily detectable shift in the pH for the maximum of f_A x f_C with pH. So if the downturn at low pH were due to protonation of A63 this should be detectable in the A63 (n7c) variant. Experimentally no significant shift was observed, so the downturn is unlikely to result from protonation of A63. The fractions f_A and f_C are calculated from:

$$f_A = \{1 + 10^{(pH - pK_a^C)}\} \Big/ \{1 + 10^{(pK_a^A - pH)} + 10^{(pK_a^A - pK_a^C)} + 10^{(pH - pK_a^C)}\}$$

$$f_C = \{1 + 10^{(pK_a^A - pH)}\} \Big/ \{1 + 10^{(pK_a^A - pH)} + 10^{(pK_a^A - pK_a^C)} + 10^{(pH - pK_a^C)}\}$$

where pK_a^A and pK_a^C are the pK_a values of A63 and C10 respectively. The three curves have been scaled so that each has a maximum value of f_A x f_C = 1.

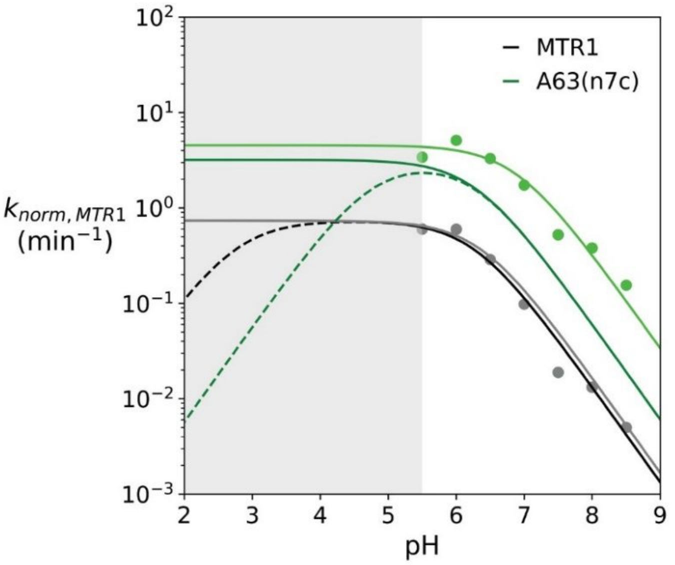

Figure S16. Comparison of the computationally constructed activity-pH profile curves obtained by single vs double pK_a models (solid and dashed lines, respectively) for MTR1 (black) and A63(n7c) (green). Experimental data points and their corresponding single pK_a fits are shown in grey and light green for MTR1 and A63(n7c), respectively. The fit parameters for the MTR1 simulated curve are k_{int} = 38.1 min^{-1}, pK_a^C = 6.3, pK_a^A = 2.8 and the parameters for the A63(n7c) simulated curves are k_{int} = 513.3 min^{-1}, pK_a^C = 6.3, pK_a^A = 4.8. A normalization factor of 0.0017 was applied to the simulated curves to overlap with experimental rates.

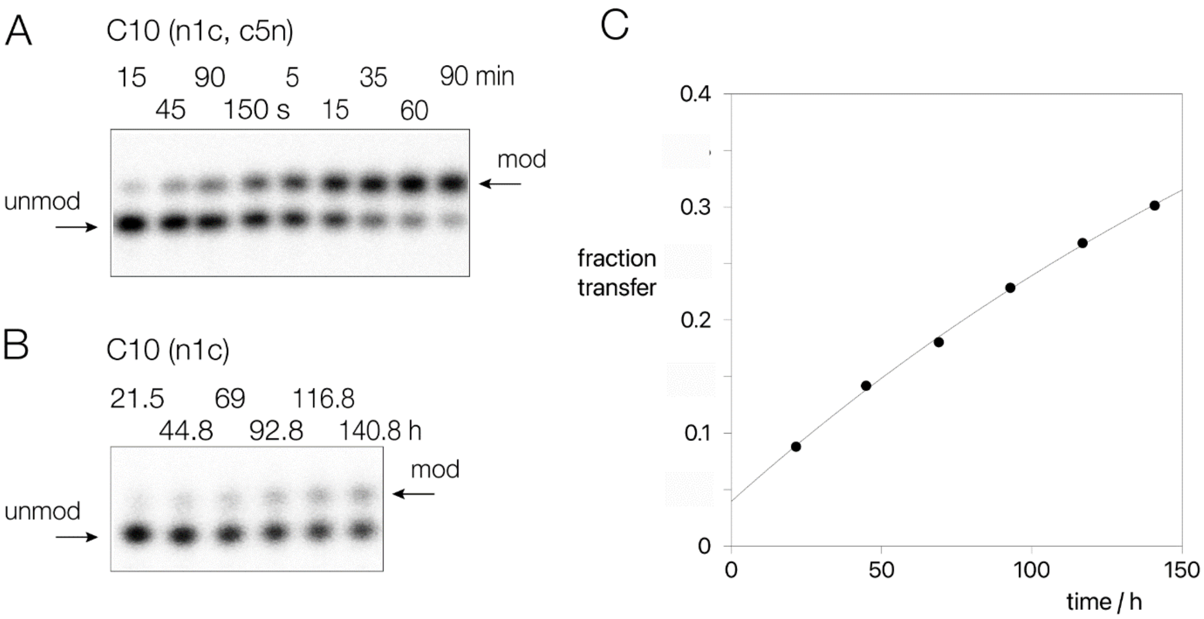

Figure S17. Time courses of alkyl transfer for the C10 (n1c, c5n) (**A**) and C10 (n1c) (**B**) variants of the MTR1 ribozyme at pH 6. The reaction times are shown above each lane. Substrate and product RNA are separated by electrophoresis in polyacrylamide gels under denaturing conditions. The unreacted RNA (unmod) migrates slightly faster than the RNA modified by 4-aminomethyl-benzyl transfer (mod) due to a combination of the additional mass and the positive charge acquired. The reactions proceed at markedly different rates – note that the time scale for MTR1 C10 (n1c, c5n) covers 90 min, whereas that for MTR1 C10 (n1c) is 6 days in duration. Reaction progress as a function of time for MTR1 C10 (n1c) is plotted in part **C**.

SUPPLEMENTARY TABLES

	pH 6.0 rate / min^{-1}	fast fraction	slow fraction	pH 7.5 rate / min^{-1}	fast fraction	slow fraction	pH 8.0 rate / min^{-1}	fast fraction	slow fraction
C10/A63	0.60	0.47	0.46	0.019	0.83	N.A.	0.013	0.95	N.A.
C10 n1c	4.4 x10^{-5}	0.98	N.A.				5.0 x10^{-5}	0.94	N.A.
C10 n1c c5n	0.67	0.44	0.43	0.06	0.28	0.60	0.0158	0.61	N.A.
A63 n7c	5.1	0.65	N.D.	0.52	0.67	N.D.	0.38	0.62	0.30

Supplementary Table S1. Measured rate constants and fractions of the fast and slow components for 4-aminomethyl-benzyl transfer for MTR1 and atomic variants at the indicated pH values. Note that the sum of the fast and slow fractions is typically ~0.9.

N.A. Not applicable - slower reactions were well fitted by a single exponential with the fraction of target RNA modified reported as the fast fraction.

N.D. Not determined – The greater separation between the rate constants for the fast and slow fractions meant that the fast rate constant could be estimated using a short reaction time with the data being well fit by a single exponential.

	Free energy (kcal·mol^{-1})					soln→ MTR1	(min^{-1})
Species	$\Delta G^{‡}_{TS1}$	ΔG_{I}	$\Delta G^{‡}_{TS2}$	ΔG_{rxn}	ΔG_a[b]	ΔpK_a	k_{int}(calc)
MTR1·ab^{6}G	6.1(8)[a]	0.5(9)	16.3(8)	-15.1(12)	2.8	2.1	431
MTR1·*O*^{6}mG	6.6(14)	0.4(8)	18.6(15)	-17.7(13)	-	-	9
C_{10}1cC·ab^{6}G	30.3(22)	1.3(21)	17.4(18)	16.0(22)	7.1	5.2	<10^{-6}
C_{10}1cC·*O*^{6}mG	30.8(26)	-0.1(37)	9.4(43)	3.2(65)	-	-	<10^{-6}
C10(n1c,c5n)·ab^{6}G	6.4(12)	0.8(8)	15.7(6)	-16.1(22)	3.9	2.9	1179
C10(n1c,c5n)·*O*^{6}mG	6.1(20)	-0.1(21)	19.5(29)	-17.7(44)	-	-	1.67
A63(n7c)·ab^{6}G	6.1(8)	0.3(6)	15.4(5)	-19.4(11)	2.9	2.1	1869
A63(n7c)·*O*^{6}mG	6.7(21)	0.6(19)	17.0(16)	-22.0(15)	-	-	132.74

Supplementary Table S2. Free energy profile data from DFTB3/3ob+ΔMLP_{PBE0} simulations of MTR1 variants with ab^{6}G and *O*^{6}mG cofactors with predicted intrinsic rates and deprotonation free energy shifts from alchemical free energy simulations.

[a] Standard errors multiplied by 100 estimated from bootstrap analysis are given in parenthesis. [b]The computed free energy of alchemically deprotonating nucleotide 10.

Species	R		TS1		I		TS2		P	
	ξ_{PT}	ξ_{AT}	ξ_{PT}	ξ_{AT}	ξ_{PT}	ξ_{AT}	ξ_{PT}	ξ_{AT}	ξ_{PT}	ξ_{AT}
MTR1·ab^{6}G	-0.81	-1.69	0.01	-1.61	0.85	-1.61	0.86	-0.24	0.85	1.30
MTR1·O^6mG	-0.86	-1.71	0.01	-1.64	0.79	-1.53	0.87	-0.22	0.88	1.49
C_{10}1cC·ab^{6}G	-0.99	-1.64	-0.88	0.03	-0.79	1.35	0.26	1.38	0.69	1.34
C_{10}1cC·O^6mG	-1.04	-1.83	-0.88	-0.03	-0.84	1.48	0.01	1.59	0.82	1.59
C10(n1c,c5n)·ab^{6}G	-0.81	-1.62	0.00	-1.73	0.75	-1.53	0.87	-0.26	0.87	1.30
C10(n1c,c5n)·O^6mG	-0.83	-1.69	-0.01	-1.66	0.77	-1.58	0.87	-0.22	0.88	1.44
A63(n7c)·ab^{6}G	-0.80	-1.70	0.02	-1.73	0.81	-1.61	0.86	-0.28	0.86	1.43
A63(n7c)·O^6mG	-0.83	-1.63	0.02	-1.55	0.8	-1.42	0.85	-0.25	0.90	1.56

Table S3. Reaction coordinates of stationary points along the reaction pathway.[a]

[a] Reaction coordinates are in units of Å. PT refers to the proton transfer coordinate and AT to the alkyl transfer coordinate.

Proton donor	cofactor	Additional metal ions	functional	ΔG‡ (kcal/mol)	k_{int}(calc) (min-1)
C10(n1c):N3	O^6mG	-	DFTB3/3ob	35.3	4.9E-12
C10(n1c):N4	O^6mG	-	DFTB3/3ob	34.8	1.1E-11
U45:N3	O^6mG	-	DFTB3/3ob	24.8	2.4E-04
A63:N6	O^6mG	-	DFTB3/3ob	33.3	1.4E-10
C10(n1c):N3	ab^6G	-	DFTB3/3ob	29.5	8.5E-08
C10(n1c):N4	ab^6G	-	DFTB3/3ob	30.3	2.3E-08
U45:N3	ab^6G	-	DFTB3/3ob	19.7	1.4E+00
A63:N6	ab^6G	-	DFTB3/3ob	30.7	1.1E-08
C10(n1c):N3	ab^6G	Na^+@C10(n1c):O2	DFTB3/3ob	27.8	1.7E-06
C10(n1c):N4	ab^6G	Na^+@C10(n1c):O2	DFTB3/3ob	27.4	2.9E-06
C10(n1c):N3	ab^6G	Mg^{2+}@A9:Rp	DFTB3/3ob	27.6	2.3E-06
C10(n1c):N4	ab^6G	Mg^{2+}@A9:Rp	DFTB3/3ob	28.0	1.0E-06
C10(n1c):N3	ab^6G	Mg^{2+}@U45:O4	DFTB3/3ob	25.6	6.2E-05
C10(n1c):N4	ab^6G	Mg^{2+}@U45:O4	DFTB3/3ob	25.8	4.7E-05
U45:N3	ab^6G	Mg^{2+}@U45:O4	DFTB3/3ob	15.2	2.7E+03
U45:N3	ab^6G	-	PBE0/6-31G*	32.6	5.0E-10
U45:N3	ab^6G	Mg^{2+}@U45:O4	PBE0/6-31G*	25.9	3.7E-05
U45:N3, C10U	ab^6G	Mg^{2+}@U45:O4	PBE0/6-31G*	28.0	1.2E-06

Table S4. Free energy barriers from C10(n1c) simulations exploring plausible proton transfer mechanisms.

References

1. Case, D. A.; Aktulga, H. M.; Belfon, K.; Ben-Shalom, I. Y.; Berryman, J. T.; Brozell, S. R.; Cerutti, D. S.; Cheatham III, T. E.; Cruzeiro, V. W. D.; Darden, T. A.; Duke, R. E.; Giambasu, G.; Gilson, M. K.; Gohlke, H.; Goetz, A. W.; Harris, R.; Izadi, S.; Izmailov, S. A.; Kasavajhala, K.; Kaymak, M. C.; King, E.; Kovalenko, A.; Kurtzman, T.; Lee, T. S.; LeGrand, S.; Li, P.; Lin, C.; Liu, J.; Luchko, T.; Luo, R.; Machado, M.; Man, V.; Manathunga, M.; Merz, K. M.; Miao, Y.; Mikhailovskii, O.; Monard, G.; Nguyen, H.; O'Hearn, K. A.; Onufriev, A.; Pan, F.; Pantano, S.; Qi, R.; Rahnamoun, A.; Roe, D. R.; Roitberg, A.; Sagui, C.; Schott-Verdugo, S.; Shajan, A.; Shen, J.; Simmerling, C. L.; Skrynnikov, N. R.; Smith, J.; Swails, J.; Walker, R. C.; Wang, J.; Wei, H.; Wolf, R. M.; Wu, X.; Xiong, Y.; Xue, Y.; York, D. M.; Zhao, S.; Kollman, P. A., AMBER22. University of California, San Francisco: San Francisco, CA, 2022.
2. Lee, T.-S.; Tsai, H.-C.; Ganguly, A.; Giese, T. J.; York, D. M., *Robust, Efficient and Automated Methods for Accurate Prediction of Protein-Ligand Binding Affinities in AMBER Drug Discovery Boost*. 2021; Vol. 1397.
3. Case, D. A.; Aktulga, H. M.; Belfon, K.; Cerutti, D. S.; Cisneros, G. A.; Cruzeiro, V. W. D.; Forouzesh, N.; Giese, T. J.; Götz, A. W.; Gohlke, H.; Izadi, S.; Kasavajhala, K.; Kaymak, M. C.; King, E.; Kurtzman, T.; Lee, T.-S.; Li, P.; Liu, J.; Luchko, T.; Luo, R.; Manathunga, M.; Machado, M. R.; Nguyen, H. M.; O'Hearn, K. A.; Onufriev, A. V.; Pan, F.; Pantano, S.; Qi, R.; Rahnamoun, A.; Risheh, A.; Schott-Verdugo, S.; Shajan, A.; Swails, J.; Wang, J.; Wei, H.; Wu, X.; Wu, Y.; Zhang, S.; Zhao, S.; Zhu, Q.; Cheatham 3rd, T. E.; Roe, D. R.; Roitberg, A.; Simmerling, C.; York, D. M.; Nagan, M. C.; Merz Jr, K. M., AmberTools. *J. Chem. Inf. Model.* **2023,** *63* (20), 6183-6191.
4. Ryckaert, J. P.; Ciccotti, G.; Berendsen, H. J. C., Numerical Integration of the Cartesian Equations of Motion of a System with Constraints: Molecular Dynamics of n-Alkanes. *SHAKE algorithm* **1977,** *23*, 327-341.
5. Loncharich, R. J.; Brooks, B. R.; Pastor, R. W., Langevin dynamics of peptides: the frictional dependence of isomerization rates of N-acetylalanyl-N'-methylamide. *Biopolymers* **1992,** *32* (5), 523-535.
6. Berendsen, H. J. C.; Postma, J. P. M.; van Gunsteren, W. F.; Dinola, A.; Haak, J. R., Molecular dynamics with coupling to an external bath. *J. Chem. Phys.* **1984,** *81*, 3684-3690.
7. Essmann, U.; Perera, L.; Berkowitz, M. L.; Darden, T.; Lee, H.; Pedersen, L. G., A smooth particle mesh Ewald method. *J. Chem. Phys.* **1995,** *103* (19), 8577-8593.
8. Darden, T.; York, D.; Pedersen, L., Particle mesh Ewald: An N· log (N) method for Ewald sums in large systems. *J. Chem. Phys.* **1993,** *98* (12), 10089-10092.
9. Petersen, H. G., Accuracy and efficiency of the particle mesh Ewald method. *J. Chem. Phys.* **1995,** *103* (9), 3668-3679.
10. Figueirido, F.; Del Buono, G. S.; Levy, R. M., On finite-size effects in computer simulations using the Ewald potential. *J. Chem. Phys.* **1995,** *103* (14), 6133-6142.
11. de Leeuw, S. W.; Perram, J. W.; Smith, E. R., Simulation of electrostatic systems in periodic boundary conditions. I. Lattice sums and dielectric constants. *Proc. R. Soc. London, Ser. A* **1980,** *373*, 27-56.
12. Nam, K.; Gao, J.; York, D. M., An efficient linear-scaling Ewald method for long-range electrostatic interactions in combined QM/MM calculations. *J. Chem. Theory Comput.* **2005,** *1* (1), 2-13.
13. Walker, R. C.; Crowley, M. F.; Case, D. A., The implementation of a fast and accurate QM/MM potential method in Amber. *J. Comput. Chem.* **2008,** *29*, 1019-1031.
14. Giese, T. J.; York, D. M., Ambient-Potential Composite Ewald Method for ab Initio Quantum Mechanical/Molecular Mechanical Molecular Dynamics Simulation. *J. Chem. Theory Comput.* **2016,** *12* (6), 2611-2632.
15. Giese, T. J.; York, D. M., A GPU-Accelerated Parameter Interpolation Thermodynamic Integration Free Energy Method. *J. Chem. Theory Comput.* **2018,** *14*, 1564-1582.

16. Warshel, A.; Levitt, M., Theoretical studies of enzymic reactions: dielectric, electrostatic and steric stabilization of the carbonium ion in the reaction of lysozyme. *J. Mol. Biol.* **1976,** *103* (2), 227-249.
17. Singh, U. C.; Kollman, P. A., A combined ab initio quantum mechanical and molecular mechanical method for carrying out simulations on complex molecular systems: Applications to the CH3Cl+ Cl− exchange reaction and gas phase protonation of polyethers. *J. Comput. Chem.* **1986,** *7* (6), 718-730.
18. Tsai, H.-C.; Lee, T.-S.; Ganguly, A.; Giese, T. J.; Ebert, M. C.; Labute, P.; Merz Jr, K. M.; York, D. M., AMBER Free Energy Tools: A New Framework for the Design of Optimized Alchemical Transformation Pathways. *J. Chem. Theory Comput.* **2023,** *19*, 640-658.
19. Deng, J.; Wilson, T. J.; Wang, J.; Peng, X.; Li, M.; Lin, X.; Liao, W.; Lilley, D. M.; Huang, L., Structure and mechanism of a methyltransferase ribozyme. *Nat. Chem. Biol.* **2022,** *18* (5), 556-564.
20. McCarthy, E.; Ekesan, Ş.; Giese, T. J.; Wilson, T. J.; Deng, J.; Huang, L.; Lilley, D. M. J.; York, D. M., Catalytic mechanism and pH dependence of a methyltransferase ribozyme (MTR1) from computational enzymology. *Nucleic Acids Res.* **2023**.
21. Scheitl, C. P. M.; Mieczkowski, M.; Schindelin, H.; Höbartner, C., Structure and mechanism of the methyltransferase ribozyme MTR1. *Nat. Chem. Biol.* **2022,** *18* (5), 547-555.
22. Hall, H. K., Jr., Correlation of the Base Strengths of Amines. *J. Am. Chem. Soc.* **1957,** *79* (20), 5441-5444.
23. Zgarbová, M.; Otyepka, M.; Šponer, J.; Mládek, A.; Banáš, P.; Cheatham III, T. E.; Jurečka, P., Refinement of the Cornell et al. nucleic acids force field based on reference quantum chemical calculations of glycosidic torsion profiles. *J. Chem. Theory Comput.* **2011,** *7*, 2886-2902.
24. Joung, I. S.; Cheatham III, T. E., Determination of alkali and halide monovalent ion parameters for use in explicitly solvated biomolecular simulations. *J. Phys. Chem. B* **2008,** *112*, 9020-9041.
25. Cieplak, P.; Cornell, W. D.; Bayly, C.; Kollman, P. A., Application of the multimolecule and multiconformational RESP methodology to biopolymers: Charge derivation for DNA, RNA, and proteins. *J. Comput. Chem.* **1995,** *16* (11), 1357-1377.
26. Gaus, M.; Goez, A.; Elstner, M., Parametrization and Benchmark of DFTB3 for Organic Molecules. *J. Chem. Theory Comput.* **2013,** *9*, 338-354.
27. Giese, T. J.; Ekesan, Ş.; McCarthy, E.; Tao, Y.; York, D. M., Surface-Accelerated String Method for Locating Minimum Free Energy Paths. *J. Chem. Theory Comput.* **2024**.
28. Shirts, M. R.; Chodera, J. D., Statistically optimal analysis of samples from multiple equilibrium states. *J. Chem. Phys.* **2008,** *129* (12).
29. Tan, Z.; Gallicchio, E.; Lapelosa, M.; Levy, R. M., Theory of binless multi-state free energy estimation with applications to protein-ligand binding. *J. Chem. Phys.* **2012,** *136*, 144102.
30. Zhang, Y., Computational investigations of HNO in biology. *J. Inorg. Biochem.* **2013,** *118*, 191-200.
31. Giese, T. J.; York, D. M., FE-ToolKit: The free energy analysis toolkit.
32. Pan, X.; Yang, J.; Van, R.; Epifanovsky, E.; Ho, J.; Huang, J.; Pu, J.; Mei, Y.; Nam, K.; Shao, Y., Machine-Learning-Assisted Free Energy Simulation of Solution-Phase and Enzyme Reactions. *J. Chem. Theory Comput.* **2021,** *17*, 5745-5758.
33. Zeng, J.; Giese, T. J.; Ekesan, Ş.; York, D. M., Development of Range-Corrected Deep Learning Potentials for Fast, Accurate Quantum Mechanical/Molecular Mechanical Simulations of Chemical Reactions in Solution. *J. Chem. Theory Comput.* **2021,** *17*, 6993-7009.
34. Giese, T. J.; Zeng, J.; Ekesan, S. o. l.; York, D. M., Combined QM/MM, Machine Learning Path Integral Approach to Compute Free Energy Profiles and Kinetic Isotope Effects in RNA Cleavage Reactions. *J. Chem. Theory Comput.* **2022,** *18* (7), 4304-4317.
35. Zeng, J.; Zhang, D.; Lu, D.; Mo, P.; Li, Z.; Chen, Y.; Rynik, M.; Huang, L. a.; Li, Z.; Shi, S.; Wang, Y.; Ye, H.; Tuo, P.; Yang, J.; Ding, Y.; Li, Y.; Tisi, D.; Zeng, Q.; Bao, H.; Xia, Y.; Huang, J.; Muraoka, K.; Wang, Y.; Chang, J.; Yuan, F.; Bore, S. L.; Cai, C.; Lin, Y.; Wang, B.; Xu, J.; Zhu, J.-

X.; Luo, C.; Zhang, Y.; Goodall, R. E. A.; Liang, W.; Singh, A. K.; Yao, S.; Zhang, J.; Wentzcovitch, R.; Han, J.; Liu, J.; Jia, W.; York, D. M.; E, W.; Car, R.; Zhang, L.; Wang, H., DeePMD-kit v2: A software package for Deep Potential models. *J. Chem. Phys.* **2023,** *159*, 054801.
36. Zhang, L.; Han, J.; Wang, H.; Saidi, W.; Car, R.; E, W., End-to-end Symmetry Preserving Inter-atomic Potential Energy Model for Finite and Extended Systems. In *Advances in Neural Information Processing Systems 31*, Bengio, S.; Wallach, H.; Larochelle, H.; Grauman, K.; Cesa-Bianchi, N.; Garnett, R., Eds. Curran Associates, Inc.: 2018; pp 4436-4446.
37. Zhang, Y.; Wang, H.; Chen, W.; Zeng, J.; Zhang, L.; Han, W.; E, W., DP-GEN: A concurrent learning platform for the generation of reliable deep learning based potential energy models. *Comput. Phys. Commun.* **2020,** *253*, 107206.
38. Lee, T.-S.; Tsai, H.-C.; Ganguly, A.; York, D. M., ACES: Optimized Alchemically Enhanced Sampling. *J. Chem. Theory Comput.* **2023,** *19*, 472-487.
39. Ganguly, A.; Tsai, H.-C.; Fernández-Pendás, M.; Lee, T.-S.; Giese, T. J.; York, D. M., AMBER Drug Discovery Boost Tools: Automated Workflow for Production Free-Energy Simulation Setup and Analysis (ProFESSA). *J. Chem. Inf. Model.* **2022,** *62* (23), 6069-6083.
40. Giese, T. J.; York, D. M., Variational Method for Networkwide Analysis of Relative Ligand Binding Free Energies with Loop Closure and Experimental Constraints. *J. Chem. Theory Comput.* **2021,** *17* (3), 1326-1336.
41. Ekesan, Ş.; McCarthy, E.; Case, D. A.; York, D. M., RNA Electrostatics: How Ribozymes Engineer Active Sites to Enable Catalysis. *J. Phys. Chem. B* **2022,** *126* (32), 5982-5990.
42. Giambaşu, G. M.; Case, D. A.; York, D. M., Predicting Site-Binding Modes of Ions and Water to Nucleic Acids Using Molecular Solvation Theory. *J. Am. Chem. Soc.* **2019,** *141* (6), 2435-2445.
